# A multicriteria analysis of meat and milk alternatives from nutritional, health, environmental, and cost perspectives

**DOI:** 10.1073/pnas.2319010121

**Published:** 2024-12-02

**Authors:** Marco Springmann

**Affiliations:** ^a^Environmental Change Institute, University of Oxford, Oxford OX1 3QY, United Kingdom; ^b^Institute for Global Health, University College London, London WC1N 1EH, United Kingdom

**Keywords:** alternative proteins, meat and milk replacements, healthy and sustainable diets, multicriteria analysis

## Abstract

Reducing meat and dairy intake has been identified as a necessary strategy for mitigating the high environmental impacts food systems are currently having on climate change, biodiversity loss associated with land-use changes, and freshwater use. Having a choice of dedicated meat and milk replacements available to consumers can help in the transition toward more plant-based diets, but concerns about nutritional and health impacts and high costs can impede uptake. Here, we conduct a multicriteria assessment of 24 meat and milk alternatives that integrates nutritional, health, environmental, and cost analyses with a focus on high-income countries. Unprocessed plant-based foods such as peas, soybeans, and beans performed best in our assessment across all domains. In comparison, processed plant-based products such as veggie burgers, traditional meat replacements such as tempeh, and plant milks were associated with less climate benefits and greater costs than unprocessed foods but still offered substantial environmental, health, and nutritional benefits compared to animal products. Our findings suggest that a range of food products exist that when replacing meat and dairy in current diets would have multiple benefits, including reductions in nutritional imbalances, dietary risks and mortality, environmental resource use and pollution, and when choosing unprocessed foods over processed ones also diet costs. The findings provide support for public policies and business initiatives aimed at increasing their uptake.

## Significance

Many meat and milk alternatives have been assessed from nutritional, environmental, and cost perspectives, but the analyses are rarely consistently combined, which limits the ability to identify cobenefits and trade-offs across domains. Our study advances each analysis and provides a comprehensive multicriteria assessment of meat and milk alternatives from nutritional, health, environmental, and cost perspectives. For a full contextualization, we compared the meat and milk alternatives to the animal products they intend to replace as well as to the unprocessed plant-based foods they are made from; we considered comparisons along multiple and complementary units of measurement, including per serving and per calorie; and we included comparisons per product as well as per overall diets in dedicated replacement analyses.

Meat and other animal products are increasingly seen as unhealthy and unsustainable consumption choices. The livestock sector is responsible for the majority of all food-related greenhouse gas (GHG) emissions and for about 20% of GHG emissions overall (1–3). In addition, animal products disproportionately affect other environmental domains, such as land use and biodiversity loss, freshwater use, and fertilizer application (4–6). Without dietary changes toward more plant-based diets, the environmental impacts of the food system are projected to pose serious challenges for efforts aimed at keeping global warming to below 2 °C (1, 7, 8) and could exceed other key planetary boundaries that attempt to define a safe operating space for humanity on a stable Earth system (1, 4).

Although animal products contain a range of important nutrients, their consumption has been linked to several chronic diseases. Consuming red meat (i.e., beef, lamb, and pork) and processed meat (obtained, e.g., through salting and curing) has been associated with increased rates of several noncommunicable diseases, including coronary heart disease, stroke, colorectal cancer, and type 2 diabetes mellitus (9–11). Based on these findings and strong mechanistic evidence, the International Agency for Research on Cancer has classified the consumption of processed meat as carcinogenic and that of red meat as probably carcinogenic (12). Although poultry and milk are seen as healthier than red meat, their consumption has been associated with significantly higher risks for stroke and coronary heart disease than equivalent plant-based foods (13–16).

Businesses are increasingly reacting to a more environmental and health-conscious public by offering a greater variety of meat and milk alternatives (17). They include products that emulate the structure of meat such as veggie burgers and veggie sausages (often based on soybeans or peas), milk replacements such as soy, oat, almond, and rice drinks, as well as traditional meat alternatives such as tofu or tempeh (both based on soybeans) (18). Efforts are also underway to produce cultivated (cellular or lab-grown) meat with promises of having lower impacts on the environment and for animal welfare than regular meat (19). In addition to private investments, public investments have been rising over the last decade and are increasingly called for, e.g., to support research and development (20).

The research on meat and milk alternatives also has been increasing, in particular since around 2018 (21, 22). The existing literature includes life cycle assessments (LCAs) quantifying the environmental impacts of meat and milk alternatives (23–25), nutritional assessments and comparisons (26–29), cost assessments and comparisons (30), and increasingly also projections of food system trajectories that include these products (31, 32). However, except for the LCA and nutritional assessments, these analyses are rarely combined in a consistent analytical framework, which limits the ability to consider the cobenefits and trade-offs across the multiple dimensions relevant for simultaneously addressing the challenges food systems are facing.

Here, we provide a comprehensive multicriteria assessment of meat and milk alternatives from nutritional, health, environmental, and cost perspectives. We included 24 food products in our assessment, ranging from traditional meat replacements such as tofu and tempeh to processed alternatives such as veggie burgers and plant-based milks, and in a sensitivity/scoping analysis also considered cultivated beef. For a full contextualization, we compared the meat and milk alternatives to the animal-source foods they intend to replace as well as to the unprocessed plant-based foods they are made from; we considered comparisons along multiple and complementary units of measurement, including per serving and per calorie; and we included comparisons per product as well as per overall diets in dedicated replacement analyses.

Each analysis of the multicriteria assessment adds unique aspects to the literature. In the nutritional analysis, we complement the usual focus on the nutritional content of foods with an analysis of how the meat and milk alternatives would impact current nutritional imbalances. In the health analysis, we developed a comparative risk assessment based on nutritional risk factors to analyze how long-term chronic disease incidence and mortality would be affected if meat and dairy were replaced with alternative products. In the environmental analysis, we integrated existing LCA estimates of GHG emissions, land use, and water use in a way that is consistent with the relevance of dietary change for mitigating resource use and pollution in those domains. In the cost analysis, we collected current supermarket data and paired them with international market surveys. Finally, in the synthesis analysis, we explored several ways of weighing up the impacts of meat and milk alternatives across the different dimensions.

## Results

Meat and milk replacements can be compared in various ways. For our main analysis, we chose two bases for comparisons. First, we compared foods based on the amounts customarily consumed. For this, we used standardized serving sizes (*SI Appendix*, Table S1) such as 110 g for most meats and alternatives, 85 g for tofu and tempeh, 240 ml for milk and alternatives, and 30 to 45 g for unprocessed foods such as pulses, nuts, and cereals. Second, we devised a replacement analysis to assess the impacts of replacing in current diets either all meat or all dairy at a calorie level. A calorie-based comparison allows for a focus on dietary and nutrient composition by controlling for differences in energy content and intake, but it also mixes foods at different servings. For example, two servings of beans contain similar calories as one serving of a veggie burger, and three servings of almond milk contain similar calories as one serving of oat or soy milk. We supplement the main analysis with a data file (https://zenodo.org/doi/10.5281/zenodo.11177060) (33) that contains comparisons based on complementary units of measurement.

As the discussion of meat and milk alternatives is taking place predominantly in high-income countries, we focused on that income region in our main analysis. We provide the results for other countries and regions in supplementary data file and discuss the key difference in a dedicated results section.

### Nutritional Analysis.

Meat and milk alternatives differ from animal-source foods both in macro- and micronutrients. For ease of comparison, we classified foods into being good (10 to 19%) or high sources (>20%) of nutrients by comparing their nutrients per serving to recommended levels of nutrient intake. While most food products covered in our analysis are good or high sources of protein, and of several minerals and vitamins (*SI Appendix*, Tables S6–S8), not all nutrients are relevant for current nutritional imbalances at a population level. Out of the 29 nutrients included in our analysis, eight had more substantial deviations (>5%) from recommended intake in high-income countries (*SI Appendix*, Table S5). They included saturated fat (60% above recommendations), calories (+7%), fiber (39% below recommendations), potassium (−36%), vitamin C (−17%), iron (−15%), riboflavin (−12%), and zinc (−10%).

**Fig. 1.**
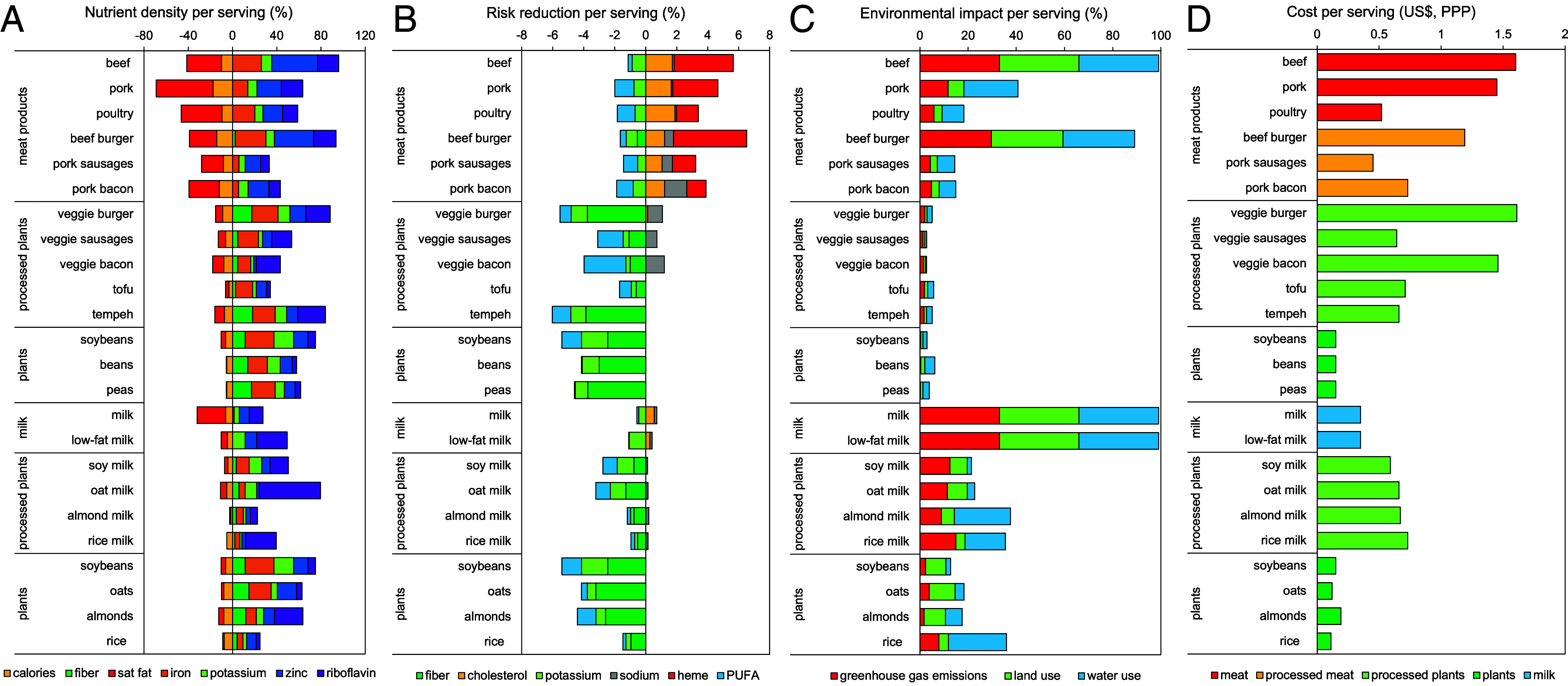
Per-serving comparison of meat and milk alternatives across nutritional (*A*), health (*B*), environmental (*C*), and cost (*D*) aspects. The nutritional comparison (*A*) displays nutritional densities (nutrients per serving as a proportion of daily recommended intake of that nutrient) for nutrients relevant to current nutritional imbalances in high-income countries. The health comparison (*B*) displays changes in overall disease risk that result from specific changes in the risks for coronary heart disease, stroke, and cancer. The environmental comparison (*C*) is expressed in relation to a benchmark food, in particular beef in the comparison of meat alternatives and milk in the comparison of milk alternatives, whose environmental impacts were normalized to 100% for the comparison. The cost comparison (*D*) displays the costs per serving of meat and milk alternatives in US Dollars adjusted for differences in purchasing power parity (PPP) across countries.

According to our analysis (Fig. 1*A*), meat products and milk had high levels (>20%) of saturated fat, and meat products had elevated levels (>10%) of calories. In comparison, meat and milk alternatives had higher levels of fiber and potassium, with elevated levels in many unprocessed alternatives. Other nutrients were comparable across products. Many or most meat products and alternatives were good sources of iron (except pork), zinc (except processed alternatives), and riboflavin (except pork and unprocessed alternatives), but no product was a good source of vitamin C. Compared to whole milk, the milk alternatives had higher amounts of iron, either higher (unprocessed alternatives) or lower (processed alternatives) amounts of zinc, either higher (processed alternatives except for almond milk) or lower (unprocessed alternatives) amounts of riboflavin, and higher (except for rice, rice milk, and almond milk) amounts of riboflavin.

**Fig. 2.**
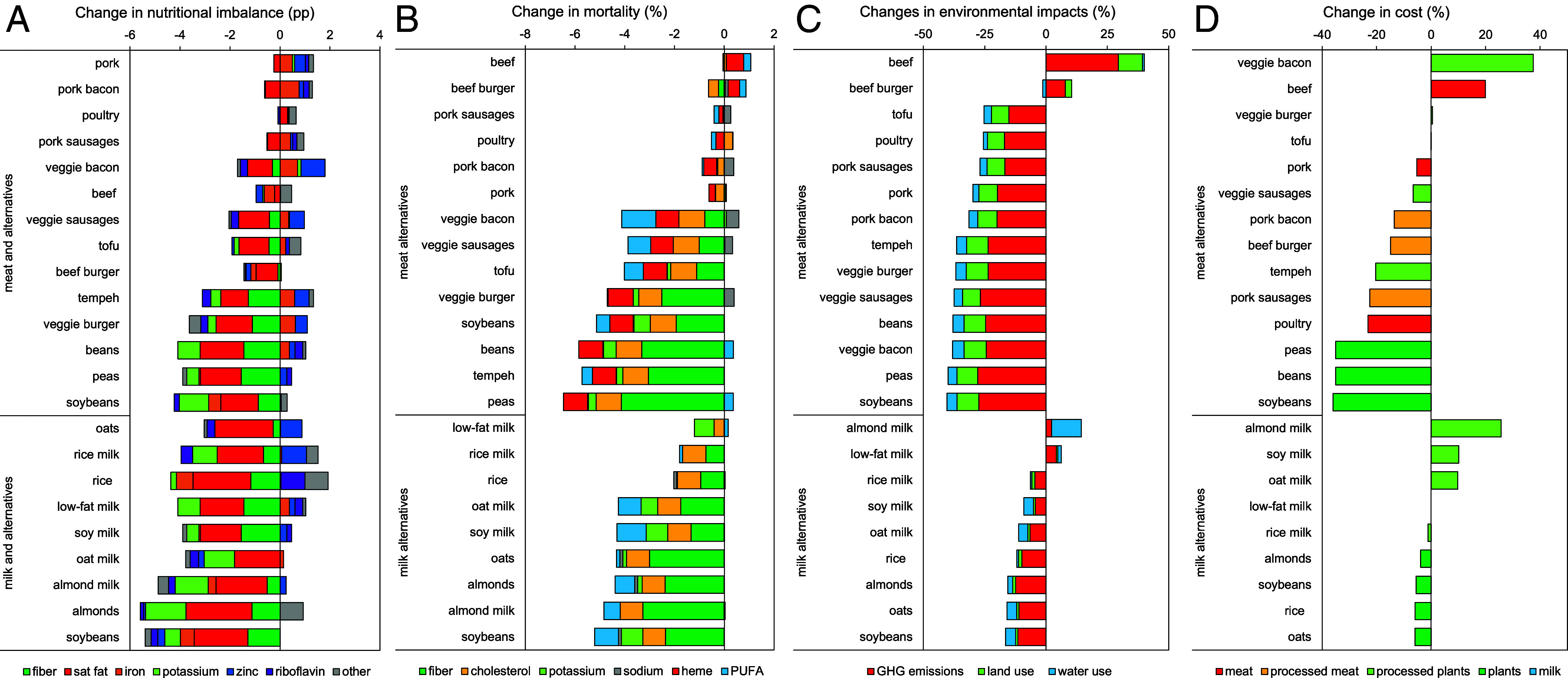
Nutritional (*A*), health (*B*), environmental (*C*), and cost (*D*) implications of replacing, per calorie, all meat in diets with meat alternatives and all dairy in diets with milk alternatives. All changes are expressed in comparison to the nutritional, health, environmental, and cost implications of current diets. Changes in nutritional imbalance are expressed in pp.

In our replacement analysis (Fig. 2*A*), replacing all calories from meat or dairy with alternatives reduced nutritional imbalances—averaged across all nutrients we assessed that had recommendations (*SI Appendix*, Table S4)—by up to 4 to 5 percentage points (pp), from 10% to 5 to 6%. Among the meat alternatives, the largest reductions were for soybeans, peas, and beans (3.1 to 4.0 pp), followed by veggie burgers (2.5 pp), tempeh (1.8 pp), and veggie sausages and tofu (1.1 pp each), while veggie bacon slightly increased imbalances (0.1 pp). Among the milk alternatives, the largest reductions were for soybeans (5.4 pp), followed by almonds and almond milk (4.6 to 4.7 pp), oat milk and soy milk (3.4 to 3.6 pp), rice and rice milk (2.4 pp each), and oats (2.2 pp). Most of the changes in nutritional imbalances were from reductions in saturated fat (41%), and from increases in fiber (20%) and potassium (12%), but they also included smaller reductions in zinc (8%), vitamin A (6%), and vitamin B12 (4%).

### Health Analysis.

While nutritional adequacy is a prerequisite for healthy diets, it is reducing dietary risk factors that are important for long-term health. High red and processed meat intake and low intake of nuts, legumes, and whole grains are important dietary risk factors that have been associated with increased risk for chronic diseases such as heart disease, stroke, and cancer (9, 10). The biological pathways responsible for the increases in risk associated with red and processed meat intake include the impacts of heme iron, cholesterol, and sodium, while those responsible for the reductions in risk associated with nuts, legumes, and whole grains include the impacts of fiber, potassium, and polyunsaturated fatty acids (PUFAs) (34–39). We constructed a comparative risk assessment based on these nutrients to compare the long-term health impacts of meats and milk with their alternatives.

According to our analysis (Fig. 1*B*), all meat and milk alternatives were associated with reductions in chronic disease risk. Among the meat alternatives, the largest reductions per serving were for tempeh and soybeans (5 to 6%), followed by peas, beans, and veggie burgers (4 to 5%), and by veggie bacon, veggie sausages, and tofu (2 to 3%). Among the milk alternatives, the largest reductions per serving were for soybeans, almonds, and oats (4 to 5%), followed by oat milk and soy milk (3%), and by rice, almond milk, and rice milk (1%). In comparison, meat products were associated with an increase in risk (2 to 5%), while milk was risk-neutral (<1%). Of the changes in risk associated with the alternatives, about half (52%) were from increases in fiber, a fifth each from increases in PUFAs (22%) and potassium (20%), and 6% from increases in sodium, while of those associated with meat, most were from increases in heme iron (42%), a quarter from increases in cholesterol (24%), a tenth each from increases in PUFAs (13%) and potassium (12%), and 8% from increases in sodium.

In our replacement analysis (Fig. 2*B*), replacing all meat or dairy in high-income countries with the same calories from meat or milk alternatives reduced mortality by up to 5 to 6%. Among the meat alternatives, the largest reductions were for peas (6.1%), followed by tempeh, beans, and soybeans (5.1 to 5.7%), veggie burgers and tofu (4.0 to 4.3%), and veggie sausages and veggie bacon (3.5 to 3.6%). Among the milk alternatives, the largest reductions were for soybeans (5.2%), followed by almond milk and almonds (4.4 to 4.8%), oats, soy milk and oat milk (4.3% each), and rice and rice milk (1.8 to 2.0%). Most of the changes in mortality were associated with increases in fiber (44%), followed by reductions in the amount of cholesterol and heme iron in diets (20% each), as well as increases in PUFAs (10%) and potassium (6%). Adding extra sodium to the unprocessed meat alternatives (peas, beans, and soybeans) or equalizing the sodium content across diets did not affect their relative ranking (*SI Appendix*, Fig. S1).

### Environmental Analysis.

Producing foods is associated with a range of environmental impacts, including GHG emissions, land use, and water use (4). For comparing the environmental impacts of meat and dairy alternatives across these domains, we collected available life-cycle assessments and, based on those, calculated environmental impact scores for each food. For this, we expressed each food’s impact as a proportion of a high-impact food (beef for meats and milk for dairy) and then averaged each food’s impacts across the three domains. For averaging, we weighted each domain by the importance dietary change has for reducing impacts in line with global environmental limits (as expressed by planetary-boundary thresholds) for that domain (1). As dietary changes are particularly important for reducing GHG emissions, this meant weighing changes in GHG emissions relatively more (at 65%) than changes in land and water use (at 17 to 18%) (*SI Appendix*, Fig. S3).

According to our analysis (Fig. 1*C*), all meat and milk alternatives had lower environmental impacts per serving than the comparable meat and milk products. Compared to beef which had the largest environmental impact among meat products (normalized to 100% across domains), beef burgers had 90% of impacts (in line with its beef content), and poultry and processed pork products had 14 to 18% of impacts (GHG: 13 to 18%, land: 9 to 10%, and water: 21 to 27%). In comparison, the impacts of meat alternatives ranged from 2% for soybeans and peas (GHG: 1%, land: 3%, and water: 5 to 8%) to 3 to 4% (GHG: 1 to 5%, land: 2 to 5%, and water: 2 to 12%) for veggie sausages, veggie bacon, and beans and 5 to 6% (GHG: 5 to 6%, land: 3 to 4%, and water: 6 to 7%) for tempeh, veggie burgers, and tofu. Compared to the average environmental impacts of whole and low-fat milk (normalized to 100% across domains), soybeans had 10% of milk’s impact (GHG: 7%, land: 25%, and water: 6%), followed by almonds and oats with 12 to 15% (GHG: 5 to 12%, land: 27 to 33%, and water: 11 to 21%), soy milk and oat milk with 28 to 29% (GHG: 35 to 38%, land: 22 to 25%, and water: 5 to 9%), rice with 31% (GHG: 24%, land: 12%, and water: 73%), almond milk with 33% (GHG: 27%, land: 16%, and water: 70%), and rice milk with 41% of impact (GHG: 45%, land: 11, and water: 51%).

Replacing all calories from meat or dairy in high-income countries with alternatives reduced the average environmental impacts by up to 40% when replacing meat and up to 16% when replacing dairy (Fig. 2*C*). Among the meat alternatives, the greatest reductions were for replacement with soybeans and peas with reductions of 40% (GHG: 42 to 43%, land: 50 to 52%, and water: 38 to 39%), followed by veggie bacon and veggie sausages with 38% (GHG: 38%, land: 52 to 53%, and water: 24 to 25%), beans, veggie burgers, and tempeh with 36 to 37% (GHG: 36 to 41%, land: 43 to 53%, and water: 17 to 24%), and tofu with 25% (GHG: 23, land: 42%, and water: 17%). Among the milk alternatives, the greatest reductions were for replacement with soybeans, oats, and almonds with reductions of 16% (GHG: 17 to 19%, land: 6 to 7%, and water: 11 to 23%), followed by rice with 12% (GHG: 15%, land: 8%, and water: 3%), oat milk and soy milk with 9 to 11% (GHG: 7 to 10%, land: 4 to 5%, and water: 21 to 22%), and rice milk with 6% (GHG: 7%, land: 7%, and water: 4%). In contrast, replacement with almond milk (of which four servings are needed to replace one serving of milk) increased average impacts by 14% (GHG: 3%, land: 0%, and water: 67%), driven by high increases in water use. Adding emissions related to processing (e.g., cooking) to the emissions footprint of unprocessed alternatives such as soybeans did not change the relative ordering (*SI Appendix*, Fig. S2).

### Cost Analysis.

High costs could present a barrier to the adoption of meat and milk alternatives. For analyzing the cost implications of meat and milk alternatives, we collected price data from British online supermarkets (40), adjusted them for differences in price levels across countries by applying purchasing power parities, and paired them with globally collected market prices that were similarly adjusted (41, 42).

According to our analysis (Fig. 1*D*), processed meat and milk alternatives were often more expensive per serving than the product they are aiming to replace, while unprocessed alternatives were less expensive. Among the meat alternatives, veggie burgers were on average 36% more expensive per serving in high-income countries than beef burgers, veggie sausages were 41% more expensive than pork sausages, and veggie bacon was 99% more expensive than pork bacon. In contrast, traditional meat alternatives were currently 41 to 45% lower in costs than beef burgers, but 26 to 36% higher in costs than poultry, while unprocessed plant-based foods (soybeans, beans, and peas) were 88% lower in costs than beef burgers and 72% lower in costs than poultry. Among the milk alternatives, soymilk was 69% more expensive per serving in high-income countries than milk, oat and almond milks were 91% more expensive, and rice milk 108% more. In contrast, the unprocessed plant-based foods used as ingredients were 45 to 69% less expensive than milk.

Replacing all calories from meat or dairy in high-income countries with alternatives decreased costs by up to 6-36% for unprocessed alternatives, and it increased cost by up to 26 to 37% for processed alternatives (Fig. 2*D*). The greatest reductions among the meat replacements were for soybeans, beans, and peas (35 to 36%), followed by tempeh (20%) and veggie sausages (7%). Replacement with tofu and veggie burgers resulted in similar costs as current diets (<1%), whereas replacement with veggie bacon increased costs by 37%. In comparison, replacement with poultry reduced costs by 23%. Among the milk replacements, the greatest reductions were for soybeans, oats, and rice (6%), followed by almonds (4%), and rice milk (1%). In contrast, replacement with soy milk or oat milk increased costs by 10%, and replacement with almond milk by 26%.

### Synthesis.

For synthesizing the performance of each food product across the different domains, we constructed a summary indicator. For each domain (nutrition, health, GHG emissions, land use, water use, and affordability), we normalized the percentage changes obtained in the replacement analyses in each domain to a scale of zero to one, with one being the best-performing food item and zero the worst. We then averaged the scores across each domain. For averaging, we considered the nutrition and mortality analyses as representing one overall health domain, assigning each a weight of one-half, and we preserved the planetary-boundary weighting of the environmental domains that assigned a greater weight to GHG emissions (0.65) than to land (0.17) and water use (0.18). Using equal weights in the environmental domain or for each subdomain resulted in similar rankings (*SI Appendix*, Fig. S5), and we mention the key differences below.

**Fig. 3.**
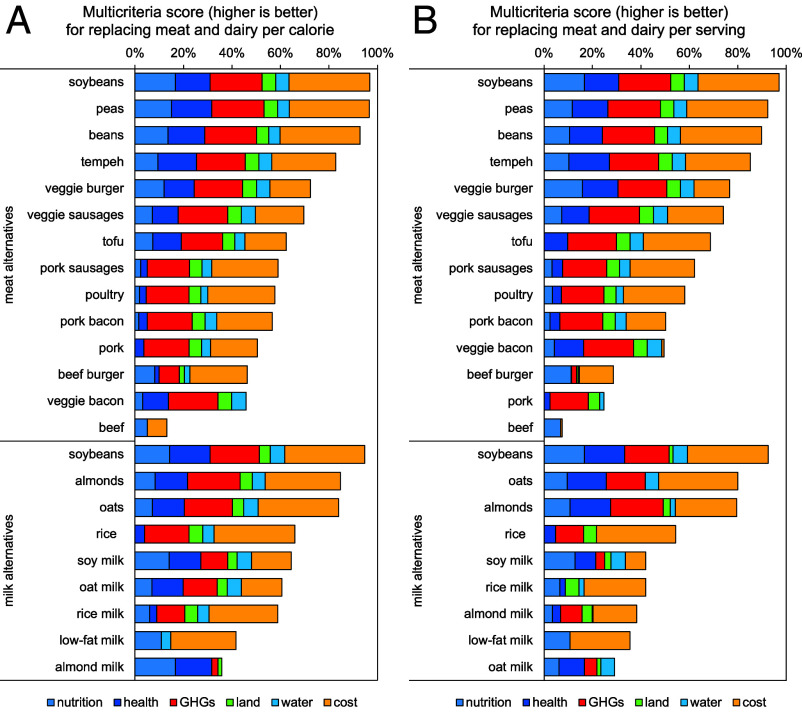
Multicriteria analysis of meat and milk alternatives. The analysis displays the synthesis scores of a food product’s overall performance in replacing meat or dairy per calorie (*A*) or per serving (*B*). The score is based on the nutritional, health, environmental, and cost changes of replacing meat and dairy with meat and milk alternatives. It gives equal weight to health, environmental, and cost impacts. The health domain includes the nutritional and mortality analyses with equal weights. The environmental domain includes analyses of changes in GHG emissions, land use, and water use, with weights assigned based on the needed contribution of dietary changes toward staying within food-related environmental limits (or planetary boundaries), which meant assigning a greater weight to changes in GHG emissions (0.65) than changes in land use (0.17) and water use (0.18).

According to our analysis (Fig. 3*A*), unprocessed plant-based foods were the best overall performers for replacing meat and dairy in high-income countries. Out of a possible summary score of 100, soybeans, peas, and beans attained scores of 93 to 97 for replacing meat, with soybeans performing best on nutrition and costs and peas best on mortality and GHG emissions. They were followed by processed plant-based products including tempeh (82), veggie burgers and veggie sausages (70–74), tofu (62), and veggie bacon (46) which performed worst on costs. In comparison, pork, poultry, and processed meats attained scores of 46 to 59, with pork performing worst on nutrition, while beef performed worst overall (13) and on health, GHG emissions, land use, and water use. Using equal weights in the environmental domain or for each subdomain resulted in similar rankings (*SI Appendix*, Fig. S5), except that the high costs per calorie of veggie bacon were penalized less in the latter. Doing the same synthesis analysis based on replacing meat by serving instead of calories resulted in a similar ranking (Fig. 3*B*), but with pork and beef burgers performing worse (25, 29) as their high calorie content did not reduce the servings required as replacement.

For replacing dairy in high-income countries, soybeans performed best overall (with a score of 95) and on mortality and water use. They were followed by almonds (85) performing best on GHG emissions, oats (84) performing best on cost, and rice (66) performing best on land use but worse on nutrition. Soy milk, oat milk, and rice milk attained scores of 59 to 65, and almond milk a score of 36 performing worst overall and on water use and costs (mainly due to its high replacement ratio stemming from its low calorie content). In comparison, low-fat milk performed second worst among dairy replacements (41) and on health, GHG emissions, and land use. Using equal weights in the environmental domain or for each subdomain resulted in similar rankings (*SI Appendix*, Fig. S5), but with plant milks attaining higher scores (63–72) as less weight was given to their high prices. Analyzing replacement per serving instead of per calorie resulted in a similar ranking (Fig. 3*B*), but with almond milk ranking better (above low-fat milk) because no increases in portions were required to compensate for its low calorie content, and oat milk ranking worse due to its relatively high cost per serving.

To illustrate the combined impacts of replacing both meat and dairy, we analyzed combinations of unprocessed and processed alternatives that performed well in their categories, including peas and soybeans in the former and veggie burgers and soy milk in the latter. In the joint replacement analysis (*SI Appendix*, Fig. S4), replacing meat and dairy with peas and soybeans at a per-calorie level reduced nutritional imbalances by 48%, mortality by 11%, food-related GHG emissions by 60%, land use by 56%, water use by 43%, and diet costs by 41%. In comparison, replacement with veggie burgers and soy milk resulted in 29% less reductions in GHG emissions (by 43%), and 23% less reductions in mortality (by 8%), absolute increases in costs (by 11%), but similar reductions in land use (58%) and water use (46%), and 33% greater improvements in nutritional imbalances (by 63%) due to complementing nutrients (in particular fortified vitamin B12).

### Regional Differences and Novel Foods.

Our main analysis focused on high-income countries as most of the discussion around meat and milk alternatives is taking place in that region. However, we did conduct the analysis by country and in aggregate also for other income regions. According to the regional assessments (*SI Appendix*, Fig. S6), the relative ranking among foods was similar in middle-income countries, with most unprocessed meat and milk alternatives ranking high, followed by most processed alternatives, and then followed by meat products and milk. A difference we identified in low-income countries was that tempeh and veggie burgers improved relatively in the ranking of meat alternatives, and oats and almonds in that of milk alternatives. This was driven predominantly by their better performance on nutrition (*SI Appendix*, Fig. S7) as nutritional deficiencies differ in low-income countries (*SI Appendix*, Table S5) and were better addressed by processed plant-based foods that are fortified and by animal-source foods.

Our main analysis focused on meat and milk alternatives that are currently available in most high-income countries. In addition to existing alternatives, so-called novel alternatives are being developed, with cultivated meat being the most prominent (19). Uncertainties regarding its impacts are high at present and, among other things, depend on the bioprocess design, the nutrient medium, and the cell types used (18, 25). Although this precludes a definite assessment, we undertook a scoping analysis to indicate a tentative ranking according to our multicriteria assessment. As an alternative to the highest-impact meat, we focused on cultivated beef and considered low and high ranges of impacts in each analysis to capture existing uncertainties (*SI Appendix*, Table S18). Although cultivating cells together with fat is still a challenge, we assumed that producing a like-for-like product is the eventual goal for companies active in this field. For the assessment, we used nutritional data for conventional beef burgers, environmental footprints from existing life-cycle assessments (43–45), and cost estimates for upscaled production systems from peer-reviewed techno-economic assessments (46–48).

In our scoping analysis (*SI Appendix*, Fig. S8), the low- and high-efficiency variants of cultivated beef ranked second and third lowest, marginally better than conventional beef (21 and 40 compared to 17), but below other meat products (48–60) and further below the plant-based alternatives (51–97). The nutritional and mortality impacts were similar to beef and beef burgers, also in a variant in which half of saturated fats were replaced by PUFAs. Land and water use were similar to plant-based alternatives, and GHG emissions ranged from being lower than poultry when produced with efficient technologies to being similar to beef burgers when produced with current technologies. The costs were the most uncertain, ranging from 40,000 times those of beef burgers when based on current processes to a factor difference of five when based on future processes and technologies that do not currently exist and are contingent on substantial technological advances and investments (46–48). Taken together, cultivated beef ranked similar to other meat products and therefore substantially lower than the processed and unprocessed alternatives made from plants. However, we stress again the high uncertainty associated with these estimates.

## Discussion

Global and regional food systems are facing multiple challenges, including how to contribute to climate change mitigation, limit the use and pollution of natural resources, contribute to adequate nutrition and good long-term health, and provide affordable food options (4). Transitioning away from diets high in meat and dairy has been identified as an essential aspect in addressing the environmental and health challenges, especially in high-income countries (1). Our analysis considered a range of meat and milk alternatives that currently exist to facilitate this transition, and contextualized them from nutritional, health, environmental, and cost perspectives. Our findings suggest that unprocessed plant-based foods such as soybeans, peas, and beans are best suited for replacing meat and dairy in high-income countries, and performed well on all dimensions. In comparison, processed plant-based foods such as veggie burgers and plant milks were associated with less climate benefits and greater costs than unprocessed foods, but still offered substantial environmental, health, and nutritional benefits compared to animal-source foods.

Our findings suggest that suitable alternatives to meat and milk exist and are available and affordable without necessarily requiring new technologies or product development. This contrasts with discussions in high-income countries on the needs to develop novel replacement foods, especially those that would completely mimic meat and dairy (18). Our nutritional, health, environmental, and cost analyses suggest that if one is prepared to consider foods for their properties instead of whether they are completely mimicking meat or dairy—and surveys suggest that consumers are (49)—then unprocessed legumes are, for the most part, superior to processed alternatives. This is also relevant for low and middle-income countries where legumes are readily available, but discussions on processed meat and milk alternatives are at an earlier stage, despite diets rapidly becoming similarly imbalanced as in high-income countries (1, 50).

Although the products we analyzed are available in many food markets, increasing their uptake might be contingent on supportive public policies and food environments. Increasing consumption of legumes, for example, could benefit from public information campaigns such as national guidelines outlining how they can form part of healthy and sustainable meals (51), alignment of agricultural subsidies (52), and business approaches that focus less on single products, but on integrating legumes in meals and diets. When it comes to processed plant-based foods, a main hurdle for their adoption are the currently high prices to consumers. Apart from scaling and improving production, a true cost approach that integrates the health and environmental costs of foods into their prices could be an option for signaling the health and environmental benefits relative to meat and dairy (53, 54). However, political economy issues could arise as the livestock sector holds considerable political influence in many markets and various interest groups aim to influence the political debate (55, 56).

The focus of our study was to be comprehensive in integrating different aspects of analysis. We analyzed 24 food products from nutritional, health, environmental, and cost perspectives. While we included most of the generic meat and milk replacements, there are many more specific products that we did not explicitly include, in part due to data availability. For example, environmental LCAs exist for different types of veggie burgers, including ones based on soy, peas, mycoprotein, and insects (23, 24). However, despite some estimates (26, 27), we were not able to obtain complete enough and independently measured nutritional data for many of these products. Increasing public investment and/or regulation to measure and report nutrition profiles that are more complete than current back-of-package labels could help us and other researchers in undertaking region-specific analyses of nutritional imbalances and long-term health.

Another challenge we faced was that LCA standards differ substantially across studies. While recent meta-analyses of LCAs attempt to harmonize the system boundaries of individual estimates by gap-filling contributions from omitted stages in the food chain (e.g., machinery manufacture, land-use changes, and handling of crop residues) (2, 57), many LCAs of the processed meat replacements that are available have not been subject to such harmonization. Reviews of LCAs indicate that environmental impacts increase with the degree of processing (25), but little with transport and packaging (2, 58). The LCAs included in our analysis mirror this trend, but the lack of harmonization of system boundaries might nevertheless mean that our estimates of the benefits of choosing unprocessed meat replacements might be underestimated.

Another aspect we did not include in our study are impacts related to changes in calorie intake and body weight. While our main replacement analysis kept calorie intake constant, the sensitivity analysis that replaced meat and dairy by serving did not. As most meat and milk alternatives are lower in calories (*SI Appendix*, Table S1), using them in a per-serving replacement would reduce energy intake and, when sustained, also body weight. Most adults in high-income countries are overweight or obese (59), so a reduction in calorie intake would, in many cases, be associated with additional health benefits. However, it is also worth noting that many of the processed meat alternatives are classified as ultraprocessed foods which have been associated with inducing overeating and weight gain in randomized controlled trails (60). Including such impacts would further improve the relative benefits of choosing unprocessed over processed meat and milk alternatives.

Finally, our analysis considered product-by-product comparisons and replacements. Although we found that unprocessed plant-based foods performed best in the multicriteria assessment, there could be trade-offs in specific domains. For example, while replacing meat with soybeans performed well from an overall nutritional perspective, it could increase nutritional deficiencies for specific nutrients that are at risk in more plant-based diets (such as vitamin B12) or fail to address existing deficiencies that neither food is a good source of (such as vitamin C). At the same time, replacing all meat and dairy with only one or two alternative foods could increase pressures on natural resource use and biodiversity in regions where those foods are currently produced. Adopting a whole diet perspective could address such issues of overreliance by diversifying replacement foods and balancing nutritional, environmental, and cost aspects at the level of diets and meals.

## Materials and Methods

We compared meat and milk alternatives across various dimensions. First, we assessed the impacts of each food product per unit of measurement, including serving size, calories, and weight. For the comparisons by serving size, we used standard reference values from the FDA’s Reference Amounts Customarily Consumed (RACC), and for the comparisons by calorie and weight, we used the nutritional information on calories per 100 g of food gathered for the nutritional analysis that is described below. In our main analysis, we first compared foods per serving size. *SI Appendix*, Table S1 provides an overview of the serving sizes and calorie contents of the foods analyzed in this study.

Second, we assessed the impacts of replacing meat and dairy with alternative products. In this analysis, we focused on the calories contained in the current intake of meat and dairy and replaced those with the same amount of calories from meat and milk alternatives. As estimates of current food intake, we used data on food availability provided by the Food and Agriculture Organization of the United Nations (FAO) and adjusted those for food wasted at the household level (61, 62). *SI Appendix*, Table S2 provides an overview of the estimates of intake used in this study. In the main analysis, we focused on replacing meat and dairy intake in high-income countries, but we provide data on other income regions and for specific countries in the Supplementary Data File (https://zenodo.org/doi/10.5281/zenodo.11177060) (33).

### Nutritional Analysis.

For the nutritional analysis, we collected nutritional data for 29 nutrients, including six proximates (calories, protein, fat, carbohydrates, sugar, and fiber), five lipids (saturated fats, monounsaturated fatty acids, polyunsaturated fatty acids, trans fats, and cholesterol), nine minerals (calcium, iron, heme iron, magnesium, phosphorus (and phytate), potassium, sodium, zinc, and copper), and nine vitamins (vitamin C, thiamine, riboflavin, niacin, pantothenate, vitamin B6, folate, vitamin B12, and vitamin A). We compiled the data on the nutritional content of foods from several databases, including USDA’s Food Data Central’s Foundation Foods database, the Food and Nutrient Database for Dietary Studies 2019 to 2020, the National Nutrient Database for Standard Reference Legacy Release, the Harvard TH Chan School of Public Health Nutrition Department’s Food Composition Table, and the regional food composition tables of the Global Expanded Nutrient Supply (GENuS) model (63). An overview of which database we used for which product is provided in *SI Appendix*, Table S3.

In the replacements analysis, we calculated changes in nutrient intake and nutritional imbalances. For calculating nutrient intake, we paired estimates of current and scenario-level food intake by food group with the associated nutrient densities. For foods other than the meat and milk replacements, we used nutrient densities extracted from regional food composition tables reported in the GENuS model (63). We then compared the estimated levels of dietary nutrient intake with a harmonized set of population-level average requirements that were based on estimates of the US National Academies of Sciences, Engineering, and Medicine and the European Food Safety Authority (EFSA) (64) and supplemented with recommendations of the World Health Organization (WHO) (65, 66). We note that requirements of population subgroups differ from the population average but are included in its calculation.

Following the nutritional recommendations, special considerations were required for some nutrients. In each replacement scenario, we adjusted zinc requirements based on the dietary phytate content and the range of phytate-dependent reference values provided by EFSA, and we adjusted iron requirements based on differentiated absorption rates of heme and nonheme iron, and an algorithm for estimating the nonheme absorption rates based on dietary mediators such as phytate, calcium, vitamin C, and meat (67). We omitted calcium from the comparison as current reference values are based on balance studies of current intake (68) and because of a lack of evidence for establishing deficient-relevant levels (69, 70). *SI Appendix*, Table S4 provides an overview of the nutrient recommendations and their sources.

Based on the nutrient-level assessments, we constructed a summary indicator to describe overall levels of nutritional imbalances and changes therein. We calculated the indicator by subtracting the percentage differences in the intake of nutrients that have maximum limits (e.g., saturated fat and sodium) from those that have minimum limits (e.g., vitamins, minerals, and fiber), in each case only for when maximum limits are exceeded or minimum limits are not met, and then dividing by the number of nutrients with recommended intake values. We calculated overall levels of nutritional imbalances in each country and aggregated these to income regions. *SI Appendix*, Table S5 provides an overview of the current nutritional imbalances calculated in this way.

### Health Analysis.

For the health analysis, we devised a comparative risk assessment based on six nutritional risk factors that have been shown to influence chronic disease incidence and mortality (71, 72). The risk factors included low intake of PUFAs, fiber, and potassium, and high intake of cholesterol, sodium, and heme iron. Relative risks that relate changes in these risk factors to changes in disease risk and mortality were adopted from meta-analyses of epidemiological cohort studies (34–39). The disease endpoints related to changes in risk factors were coronary heart disease (CHD), stroke, and cancer. *SI Appendix*, Table S9 provides an overview of the relative risks used in the assessment. In line with the epidemiological evidence, we capped the maximally attainable benefits of potassium intake at 3,500 mg (36). *SI Appendix*, Table S10 provides an overview of risk exposures from dietary intake in the replacement scenarios.

We estimated the mortality and disease burden attributable to the nutritional risk factors by calculating population impact fractions (PIFs) based on the relative risks and risk exposure levels, and then applying those to cause-specific mortality rates and population numbers in a country and region (73–75). PIFs represent the proportions of disease cases that would be avoided when the risk exposure was changed from a baseline situation (the benchmark diet) to a counterfactual situation (the dietary replacement scenarios). For these calculations, we adopted mortality and population data from the Global Burden of Disease project (76). *SI Appendix*, Tables S11 and S12 provide an overview of the estimates of attributable deaths by risk factor and cause for the different replacement scenarios.

### Environmental Analysis.

For the environmental analysis, we used estimates from life cycle assessments (LCAs) for GHG emissions, land use, and fresh/bluewater use. LCAs track the environmental impacts of food products across the food chain, including production, processing, transport, and retail (77). Where possible, we used regionalized values from meta-analyses of LCAs that harmonized input data and scope of assessments from farm to retail (2). This was possible in particular for the unprocessed foods covered by our study. For processed meats and plant-based products, we used a combination of peer-reviewed LCAs and LCAs commissioned by industry, giving priority to peer-reviewed LCAs where possible. Out of the eight processed products analyzed in this study, two had data from peer-reviewed LCAs (veggie burgers and tempeh) (29, 78–80), two from meta-analyses of LCAs (beef burger with 90% beef content and tofu) (2, 57), three from industry LCAs (pork sausages, pork bacon, and veggie bacon) (81, 82), and one a combination of peer-reviewed LCAs and industry LCAs (veggie sausages) (78, 81, 82). *SI Appendix*, Table S13 provides an overview of the footprints data and sources used in the assessment.

For the replacement analysis, we paired the consumption scenarios with environmental footprints at serving and calorie levels. We converted the footprints reported by weight to serving and calorie bases by using data on serving sizes and calorie content collected for the nutritional analysis (*SI Appendix*, Tables S1 and S3). For calculating the environmental impacts of current food intake, we supplemented the footprints of the products assessed in this study by environmental footprints for the remainder of foods that make up diets from the same meta-analysis of LCAs that we utilized for unprocessed foods (2). We note that the impacts of soybeans are for those intended for human consumption, which do not have the same emissions from land-use changes as those intended for animal feed (2, 57). *SI Appendix*, Table S14 provides an overview of baseline and scenario impacts calculated in that way.

### Cost Analysis.

For the cost analysis, we used market price data. We collected market prices for meat and milk alternatives from UK online supermarkets and paired them with internationally collected market prices. To ensure comparability, we first converted both sets of data to a common year (2020) by using values of the consumer price index (CPI) for food (adopted from the OECD), which adjusts for inflation across years. We then converted national currency values to international dollars using PPP rates (also adopted from the OECD), which controls for price differences across countries.

For unprocessed foods and for calculating changes in the cost of diets in the replacement analysis, we paired the supermarket data with international price data from the International Comparison Program (ICP) of the World Bank (42). In previous research, we aggregated the ICP data (covering 463 food items with 20,666 estimates of annual average prices in 179 countries) to a list of 31 food groups commonly used to construct diet scenarios (41). For the aggregation, we paired each item with its caloric content (sourced from USDA’s FoodData Central database) to control for differences in processing and edible fractions, and we converted averaged prices from local currency to international dollars based on PPP rates that control for differences in price levels across countries. *SI Appendix*, Table S15 provides an overview of the price data we used in the analysis, and *SI Appendix*, Table S16 lists the estimated costs of diets for the baseline and the replacement scenarios.

### Synthesis.

For synthesizing the results of the nutritional, health, environmental, and cost analyses, we devised a multicriteria analysis that summarizes the performance of each food product across the different domains. For that, we used the results of the replacement analysis and normalized the percentage changes in each domain (e.g., reduction in nutritional imbalances, diet-related mortality, environmental impacts, and costs) to a scale of zero to one, with zero the worst-performing product and one the best-performing one. For normalizing the impacts, we first subtracted the percentage changes in each domain by the best-performing food product (giving the best-performing product a value of zero), then divided by the worst-performing item (giving the worst-performing product a value of one), and finally subtracted the results from one (inverting the scaling with one assigned to the best performing and zero to the worst performing).

We calculated a weighted average across the domains to obtain an overall summary indicator. In line with the overall domains of the assessment, we equally weighed the overall health, environmental, and cost domains. In the health domain, we assigned equal weights to the nutrition and mortality analysis. In the environmental domain, we weighed each subdomain in line with the percentage contributions that dietary changes were estimated to need to make to stay within planetary boundaries (*SI Appendix*, Fig. S3) (1), which meant assigning greater weights to changes in GHG emissions (0.65) than for land use (0.17) and water use (0.18). No subdomain weighing was needed in the cost domain. *SI Appendix*, Table S17 provides an overview of the unweighted scores in each subdomain, and *SI Appendix*, Fig. S5 compares the main weighting scheme with equal weights in the environmental domain and with equal weights for each subdomain.

## Supplementary Material

Appendix 01 (PDF)

## Data Availability

XLSX data have been deposited in Zenodo (https://zenodo.org/doi/10.5281/zenodo.11177060) (33).
