# Supplementary material for: A multicriteria analysis of meat and milk alternatives from nutritional, health, environmental, and cost perspectives
Source: Proc Natl Acad Sci U S A. 2024 Dec 2;121(50):e2319010121. doi: 10.1073/pnas.2319010121 (PMC11648608; doi:10.1073/pnas.2319010121)

Supporting Information for

A multi-criteria analysis of meat and milk alternatives from nutritional, health, environmental, and cost perspectives

Marco Springmann[1,2]*
[1]Environmental Change Institute, University of Oxford, Oxford, UK
[2]Centre on Climate Change and Planetary Health, London School of Hygiene and Tropical Medicine, London, UK

*Email: marco.springmann@ouce.ox.ac.uk; marco.springmann@lshtm.ac.uk

This PDF file includes:

Figures S1 to S8
Tables S1 to S18

Supplementary nutritional data and results

Table S1. Comparison of food products by serving sizes and calories.

Category	Food product	Serving size (g)	Calories per serving	Calories per 100 g	Servings (g) per 200 kcal
meat	beef	110	222	202	99
	pork	110	388	352	57
	poultry	110	211	192	104
	beef burger	110	317	288	69
	pork sausages	55	179	325	62
	pork bacon	55	266	484	41
meat alternative	veggie burger	110	195	177	113
	veggie sausages	55	140	255	78
	veggie bacon	55	170	309	65
	tofu	85	71	83	241
	tempeh	85	163	192	104
	soybeans	35	139	396	50
	beans	35	116	332	60
	peas	35	116	331	61
milk	milk	240	138	58	348
	low-fat milk	240	103	43	465
milk alternative	soy milk	240	91	38	526
	oat milk	240	115	48	417
	almond milk	240	36	15	1333
	rice milk	240	113	47	426
	soybeans	35	139	396	50
	oats	45	171	380	53
	almonds	30	173	576	35
	rice	45	166	368	54

Table S2. Overview of food intake and availability in high-income countries in 2020.

Food group	Food availability		Food intake	
	(g/d)	(kcal/d)	(g/d)	(kcal/d)
wheat	232	662	135	384
rice	61	165	47	128
maize	29	80	15	42
other grains	21	55	12	31
roots	140	94	99	66
sugar	129	417	74	241
legumes	11	37	10	35
soybeans	4	13	4	13
nuts&seeds	20	79	15	60
vegetable oils	49	506	47	481
palm oil	3	38	3	27
vegetables	294	78	180	48
tropical fruits	79	35	48	22
temperate fruits	121	74	74	45
starchy fruits	31	19	19	12
beef	61	72	37	44
lamb	7	15	5	9
pork	86	183	50	106
poultry	98	137	62	85
eggs	39	55	34	48
milk	551	317	493	283
shellfish	24	10	11	4
freshwater fish	14	9	6	4
pelagic fish	18	20	8	9
demersal fish	20	18	9	8
other crops	261	321	155	226
total	2,403	3,508	1,649	2,460

Table S3. Overview of data sources of nutritional data.

Category	Food product	Data source
meat	beef	GENuS; Harvard FCT
	pork	GENuS; Harvard FCT
	poultry	GENuS; Harvard FCT
	beef burger	Survey (FNDDS), FDC ID: 27510601
	pork sausages	Survey (FNDDS), FDC ID: 2341603
	pork bacon	Survey (FNDDS), FDC ID: 2341293
meat alternative	veggie burger	Survey (FNDDS), FDC ID: 2342948
	veggie sausages	SR Legacy, FDC ID: 174269
	veggie bacon	Survey (FNDDS), FDC ID: 2342940
	tofu	SR Legacy, FDC ID: 174290
	tempeh	SR Legacy, FDC ID: 174272
	soybeans	GENuS; Harvard FCT
	beans	GENuS; Harvard FCT
	peas	GENuS; Harvard FCT
milk	milk	GENuS; Harvard FCT
	low-fat milk	Foundation, FDC ID: 746772
milk alternative	soy milk	Foundation, FDC ID: 1999630
	oat milk	Foundation, FDC ID: 2257046
	almond milk	Foundation, FDC ID: 1999631
	rice milk	SR Legacy, FDC ID: 171942
	soybeans	GENuS; Harvard FCT
	oats	GENuS; Harvard FCT
	almonds	GENuS; Harvard FCT
	rice (brown)	Foundation, FDC ID: 2512380

Abbreviations: GENuS denotes the Global Expanded Nutrient Supply model; Harvard FCT: Harvard University Food Composition Tables; FDC: USDA's Food Data Central; FNDDS: Food and Nutrient Database for Dietary Studies; SR Legacy: Standard Reference Legacy Release; Foundation: USDA's Food Data Central's Foundation Foods.

Notes: Where nutrient data were adapted from GENuS and the Havard Food Comoposition Tables (FCT), the split was such that data for pantothenate, vitamin B12, and cholesterol were sourced from the Harvard FCT, and the rest from GENuS.

Table S4. Overview of nutrient recommendations and their sources.

Nutrient	Unit	Recommendation	Min, max, or mean	Source
calories	kcal	2212.9	mean	NASEM
protein	g	46.0	min	EFSA
fiber	g	31.0	min	NASEM
saturated fats	g	24.6	max	WHO
polyunsaturated fats	g	14.8	min	WHO
iron	mg	11.6	min	EFSA
magnesium	mg	276.2	min	NASEM
phosphorus	mg	592.1	min	NASEM
potassium	mg	3358.8	min	WHO
sodium	mg	2000.0	max	WHO
zinc	mg	9.7	min	EFSA
copper	mg	0.7	min	NASEM
vitamin C	mg	77.3	min	EFSA
thiamine	mg	0.9	min	NASEM
riboflavin	mg	1.2	min	EFSA
niacin	mg	10.7	min	NASEM
pantothenate	mg	3.9	min	EFSA
vitamin B6	mg	1.3	min	EFSA
folate	μg	235.3	min	EFSA
vitamin B12	μg	1.9	min	NASEM
vitamin A	μg	498.2	min	EFSA

Abbreviations: NASEM: US National Academy of Sciences, Engineering, and Medicine; EFSA: European Food Safety Authority; WHO: World Health Organization.

Notes: All recommendations were regionalised depending on the age and sex composition of a country's population. The recommendations for iron depend on estimates of absorption that are re-calculated in each scenario. The recommendations for zinc depend on the phytate content of diets and is also re-calculated in each scenario.

Table S5. Overview of nutritional imbalance (percentage difference to recommended intake) by income region, including high-income (HIC), upper middle-income (UMC), lower middle-income (LMC), and low-income (LIC) countries. The values are population-weighted averages of imbalances in each country. Negative values denote imbalanced intake and also applies to when maximum recommended intakes are exceeded (i.e., for calories, saturated fats, sodium). The average nutritional imbalance was calculated by subtracting the percentage differences in the intake of nutrients that have maximum limits (e.g., saturated fats and sodium) from those that have minimum limits (e.g., vitamins, minerals, and fiber), in each only for when maximum limits are exceeded or minimum limits are not met, and then dividing by the number of nutrients with recommended intake values.

Category	Nutrients	Nutritional imbalance (%)			
		HIC	UMC	LMC	LIC
Proximates and lips	calories	8.6	6.2	4.4	-11.6
	protein	0.0	0.0	0.0	0.0
	fiber	-39.0	-17.5	-36.5	-13.0
	saturated fats	60.4	10.6	5.2	0.0
	polyunsaturated fats	-0.1	-2.8	-12.1	-34.1
Minerals	iron	-13.7	-3.5	-2.7	-1.6
	magnesium	-2.3	-4.4	-1.2	-1.5
	phosphorus	0.0	0.0	0.0	-0.2
	potassium	-34.0	-14.8	-34.2	-17.8
	sodium	0.0	0.0	0.0	0.0
	zinc	-7.6	-9.2	-16.2	-25.2
	copper	0.0	0.0	0.0	0.0
Vitamins	vitamin C	-16.4	-5.0	-19.7	-21.4
	thiamin	-1.7	-0.3	-7.9	-1.9
	riboflavin	-9.9	-7.1	-45.6	-29.1
	niacin	-0.3	-0.1	-0.2	-0.8
	pantothenate	0.0	-0.2	-0.6	-4.1
	vitamin B6	-0.7	-0.6	-0.1	-1.0
	folate	-4.7	-4.4	-3.0	-3.0
	vitamin B12	0.0	-0.6	-18.6	-44.0
	vitamin A	-3.6	-1.2	-22.0	-10.5
Average nutritional imbalance		-9.7	-4.2	-11.0	-10.5

Table S6. Classification of food products into good (10-19%, orange-shaded) and high (>20%, red-shaded) sources of macronutrients.

Category	Food product	Nutrient content per serving (% of recommended intake)				
		calories	protein	fiber	saturated fats	polyunsat fats
meat	beef	10.0	43.7		31.2	5.3
	pork	17.5	33.8		51.5	26.0
	poultry	9.6	45.1		36.8	23.7
	beef burger	14.3	41.9	2.5	24.6	8.4
	pork sausages	8.1	22.1		19.8	19.1
	pork bacon	12.0	44.7		27.5	22.6
meat alternative	veggie burger	8.8	37.5	17.4	6.4	15.1
	veggie sausages	6.3	24.3	5.0	6.6	34.6
	veggie bacon	7.7	14.0	4.6	10.3	57.4
	tofu	3.2	18.4	2.7	3.2	15.3
	tempeh	7.4	37.5	18.1	8.8	24.8
	soybeans	6.3	26.5	11.4	4.1	26.4
	beans	5.3	16.9	13.7	0.3	1.0
	peas	5.2	16.4	17.3	0.2	1.2
milk	milk	6.2	17.0		25.6	2.7
	low-fat milk	4.7	17.6		5.5	0.5
milk alternative	soy milk	4.1	18.5	3.5	3.1	18.7
	oat milk	5.2	4.2	5.8	5.5	19.1
	almond milk	1.6	2.9	3.5	1.0	4.5
	rice milk	5.1	1.5	2.3		5.1
	soybeans	6.3	26.5	11.4	4.1	26.4
	oats	7.7	15.7	14.9	2.2	7.7
	almonds	7.8	13.9	12.0	4.7	25.0
	rice	7.5	7.1	4.4	1.3	3.8

Table S7. Classification of food products into good (10-19%, orange-shaded) and high (>20%, red-shaded) sources of minerals.

Category	Food product	Nutrient content per serving (% of recommended intake)						
		iron	mag-nesium	phos-phorus	potassium	sodium	zinc	copper
meat	beef	26.0	6.9	32.8	9.3	3.9	41.7	16.2
	pork	13.9	5.2	29.3	8.3	3.1	22.0	9.8
	poultry	20.3	7.8	29.8	7.4	3.9	17.5	14.4
	beef burger	27.9	8.4	26.6	7.5	19.5	35.4	15.2
	pork sausages	5.9	3.2	13.8	5.6	22.4	13.9	6.0
	pork bacon	5.5	6.8	37.3	8.7	48.4	18.7	9.9
meat alternative	veggie burger	23.8	22.3	38.3	10.9	31.3	14.2	33.9
	veggie sausages	18.3	7.2	20.9	3.8	24.4	8.3	21.2
	veggie bacon	11.9	3.8	6.5	2.8	40.2	2.4	8.9
	tofu	15.5	10.8	15.9	3.3	0.2	9.4	26.2
	tempeh	20.6	24.9	38.2	10.4	0.4	10.0	73.2
	soybeans	26.0	35.1	39.7	18.0	0.1	12.8	87.5
	beans	17.7	21.4	22.7	11.7	0.3	10.8	45.5
	peas	21.4	8.8	18.0	8.2	0.3	9.8	47.9
milk	milk	1.7	10.3	29.6	4.6	5.4	8.8	10.4
	low-fat milk		10.4	41.8	11.4	4.7	10.6	0.4
milk alternative	soy milk	11.6	18.7	28.0	11.3	4.1	7.7	39.9
	oat milk	5.6	5.1	36.1	10.6	5.0	2.2	10.0
	almond milk	6.2	5.9	12.2	2.2	7.2	4.2	7.8
	rice milk	4.3	9.6	22.7	1.9	4.7	3.2	13.7
	soybeans	26.0	35.1	39.7	18.0	0.1	12.8	87.5
	oats	20.1	28.8	37.6	5.7	0.2	17.4	43.3
	almonds	9.6	29.4	24.4	6.6	0.0	10.0	47.6
	rice	5.0	18.7	23.0	3.4	0.1	8.6	18.4

Table S8. Classification of food products into good (10-19%, orange-shaded) and high (>20%, red-shaded) sources of vitamins.

Category	Food product	Nutrient content per serving (% of recommended intake)								
		vitamin C	thiamine	riboflavin	niacin	panto-thenate	vitamin B6	folate	vitamin B12	vitamin A
meat	beef	1.6	9.9	19.0	40.6	15.6	27.0	3.9	129.0	2.4
	pork	1.5	48.0	19.4	40.5	20.6	22.8	1.8	39.2	2.3
	poultry	1.8	8.0	13.8	54.8	25.7	30.3	3.8	18.4	7.7
	beef burger	0.7	30.3	20.3	49.7		23.7	23.8	96.9	
	pork sausages		16.0	7.9	31.5		8.3	0.2	29.1	3.1
	pork bacon		35.0	10.5	54.0		22.6		32.4	1.2
meat alternative	veggie burger	6.4	33.1	22.0	38.6	8.2	25.6	58.0	119.5	0.2
	veggie sausages		14.6	18.1	57.6	4.6	35.0	6.1		
	veggie bacon		27.5	21.7	38.9		20.3	9.8		0.4
	tofu		4.6	3.3	1.9	18.4	5.4	3.3		
	tempeh		7.5	24.9	21.0	6.1	14.1	8.7	3.7	
	soybeans	2.5	19.5	7.0	5.7	7.1	11.0	55.4		1.9
	beans	0.3	17.2	3.9	6.2	6.7	8.4	50.3		0.3
	peas	0.4	15.7	5.0	9.9		4.7	24.8		0.9
milk	milk	1.9	3.6	12.5	1.9	22.8	11.3	3.9	58.4	17.2
	low-fat milk		15.6	27.5	2.5	22.7	11.1	2.0	79.1	27.9
milk alternative	soy milk		17.2	16.5	5.3		10.2	20.4	50.6	27.9
	oat milk		10.9	55.3	2.2		1.1		66.2	41.0
	almond milk		3.0	6.5	1.7		1.9	6.1	44.1	19.8
	rice milk		7.4	27.9	8.8	9.0	7.2	2.0	81.7	29.9
	soybeans	2.5	19.5	7.0	5.7	7.1	11.0	55.4		1.9
	oats		35.6	4.9	4.2	13.0	4.1	10.7		0.0
	almonds	1.2	6.5	25.5	10.5		3.2	5.6		
	rice		16.7	3.8	26.4					

Supplementary health data and results

Table S9. Relative risk values used in the mortality analysis, including mean values and low and high values of 95% confidence intervals.

Risk factor	Endpoint	Unit	Relative risk			Source
			mean	low	high	
fiber	CHD	per 10 g	0.89	0.85	0.93	Kim and Je (2016)
fiber	Cancer	per 10 g	0.94	0.91	0.97	Kim and Je (2016)
potassium	Stroke	at 3500 mg	0.67	0.57	0.78	Vinceti et al (2016)
PUFA	CHD	per 5% E	0.90	0.85	0.94	Farvid et al (2014)
heme	CHD	per 1 mg	1.07	1.01	1.14	Fang et al (2015)
heme	Stroke	per 1 mg	1.07	1.01	1.14	Fang et al (2015)
cholesterol	Cancer	per 100 mg	1.06	1.05	1.07	Mofrad et al (2022)
sodium	Stroke	per 1 g	1.06	1.02	1.10	Jayedi et al (2019)

Table S10. Overview of exposure of dietary risks in the baseline and the per-calorie replacement scenarios.

Category	Food product	Exposire levels by risk factor					
		fiber	cholesterol	potassium	sodium	heme	PUFA
baseline		19.14	276.12	2,153.76	463.58	0.65	13.87
meat	beef	19.14	278.79	2,198.18	474.52	1.04	13.07
	pork	19.14	253.62	2,089.27	434.70	0.48	13.78
	poultry	19.14	296.07	2,164.19	484.24	0.43	14.40
	beef burger	19.73	249.34	2,110.00	703.72	0.90	13.10
	pork sausages	19.14	272.48	2,171.13	1,015.22	0.53	14.41
	pork bacon	19.14	257.77	2,180.31	1,292.38	0.31	14.05
meat alternative	veggie burger	25.92	214.60	2,368.21	1,189.12	0.03	13.93
	veggie sausages	21.82	207.68	2,136.33	1,254.67	0.03	16.68
	veggie bacon	21.20	207.68	2,051.77	1,558.96	0.03	18.16
	tofu	22.09	207.68	2,293.31	413.66	0.03	16.20
	tempeh	27.55	207.68	2,431.08	413.33	0.03	15.14
	soybeans	24.38	207.68	2,897.78	403.05	0.03	15.50
	beans	28.12	207.68	2,727.91	414.87	0.03	12.80
	peas	30.50	207.68	2,489.70	413.78	0.03	12.83
milk	milk	19.14	276.12	2,153.76	463.58	0.65	13.87
	low-fat milk	19.14	251.53	2,985.96	472.79	0.65	13.47
milk alternative	soy milk	22.49	218.58	3,084.07	469.32	0.65	17.23
	oat milk	23.56	218.58	2,841.07	463.73	0.65	16.50
	almond milk	27.64	218.58	2,568.89	1,349.29	0.65	15.72
	rice milk	20.95	218.58	2,158.20	450.92	0.65	14.22
	soybeans	25.19	218.58	3,070.27	217.18	0.65	16.64
	oats	26.80	218.58	2,306.60	222.67	0.65	14.22
	almonds	25.23	218.58	2,352.24	216.78	0.65	16.09
	rice	21.45	218.58	2,187.08	217.70	0.65	13.80

Notes: Units are g for fiber, mg for cholesterol, mg for potassium, mg for sodium, mg for heme, and percent of daily energy for PUFA.

Table S11. Number of attributable deaths in high-income countries by risk factor for replacing all meat and dairy calories with meat and milk alternatives.

Category	Food product	Attributable deaths by risk factor						
		all risks	fiber	cholesterol	potassium	sodium	heme	PUFA
meat	beef	114,344	32	9,178	-5,672	834	77,458	32,514
	pork	-58,192	32	-38,855	6,861	-1,508	-27,998	3,276
	poultry	-18,638	32	38,544	-1,464	1,148	-37,173	-19,726
	beef burger	27,545	-26,165	-45,017	4,505	12,858	51,030	30,333
	pork sausages	-16,543	32	-4,072	-2,007	28,839	-18,080	-21,254
	pork bacon	-56,167	32	-30,262	-2,810	41,990	-57,735	-7,382
meat alternative	veggie burger	-480,644	-281,031	-103,663	-24,468	44,680	-113,565	-2,597
	veggie sausages	-396,528	-113,346	-116,231	2,153	34,620	-101,199	-102,526
	veggie bacon	-395,990	-87,150	-116,481	9,972	55,395	-104,197	-153,529
	tofu	-449,578	-124,242	-116,137	-14,632	-2,544	-106,465	-85,558
	tempeh	-640,312	-341,421	-114,951	-28,502	-2,543	-105,836	-47,059
	soybeans	-574,793	-216,657	-115,503	-73,920	-3,035	-105,661	-60,017
	beans	-613,281	-371,801	-114,877	-57,413	-2,446	-108,572	41,828
	peas	-682,638	-463,120	-114,619	-34,307	-2,515	-108,577	40,499
milk	milk	0	0	0	0	0	0	0
	low-fat milk	-115,790	0	-45,508	-88,290	785	0	17,224
milk alternative	soy milk	-480,890	-149,637	-103,791	-97,290	590	0	-130,762
	oat milk	-476,157	-195,814	-103,521	-74,125	276	0	-102,974
	almond milk	-538,479	-365,909	-102,877	1,865	1,491	0	-73,048
	rice milk	-201,921	-82,787	-104,326	-25	-442	0	-14,342
	soybeans	-582,913	-263,856	-103,215	-95,031	-13,237	0	-107,574
	oats	-483,008	-336,308	-102,977	-16,538	-12,994	0	-14,190
	almonds	-491,706	-266,426	-103,201	-21,505	-13,316	0	-87,258
	rice	-223,406	-105,856	-104,129	-3,293	-13,348	0	3,219

Table S12. Number of attributable deaths in high-income countries by cause for replacing all meat and dairy calories with meat and milk alternatives.

Category	Food product	Attributable deaths by cause			
		all causes	CHD	stroke	cancer
meat	beef	114,344	85,925	19,227	9,191
	pork	-58,192	-15,142	-4,208	-38,842
	poultry	-18,638	-44,320	-12,876	38,558
	beef burger	27,545	50,186	33,621	-56,262
	pork sausages	-16,543	-33,214	20,730	-4,058
	pork bacon	-56,167	-46,774	20,855	-30,248
meat alternative	veggie burger	-480,644	-231,817	-21,622	-227,205
	veggie sausages	-396,528	-236,427	6,168	-166,269
	veggie bacon	-395,990	-272,641	31,634	-154,984
	tofu	-449,578	-225,677	-52,950	-170,951
	tempeh	-640,312	-306,698	-66,588	-267,026
	soybeans	-574,793	-251,065	-112,129	-211,599
	beans	-613,281	-241,395	-95,139	-276,747
	peas	-682,638	-292,994	-72,295	-317,349
milk	milk	0	0	0	0
	low-fat milk	-115,790	17,224	-87,505	-45,508
milk alternative	soy milk	-480,890	-215,605	-96,700	-168,585
	oat milk	-476,157	-213,843	-73,849	-188,466
	almond milk	-538,479	-279,200	3,356	-262,635
	rice milk	-201,921	-61,831	-467	-139,624
	soybeans	-582,913	-256,314	-108,268	-218,331
	oats	-483,008	-205,999	-29,533	-247,476
	almonds	-491,706	-237,765	-34,820	-219,120
	rice	-223,406	-57,721	-16,640	-149,045

Fig. S1. Sensitivity analysis of replacing meat per calorie with meat alternatives – with salt added to unprocessed plant-based foods (left) and with salt content equalized across replacement scenarios (right). For the analysis shown on the left, one serving of salt (590 mg sodium) was added per 100 g of peas, beans, and soybeans in the scenarios of replacing all meat by calories with meat alternatives.

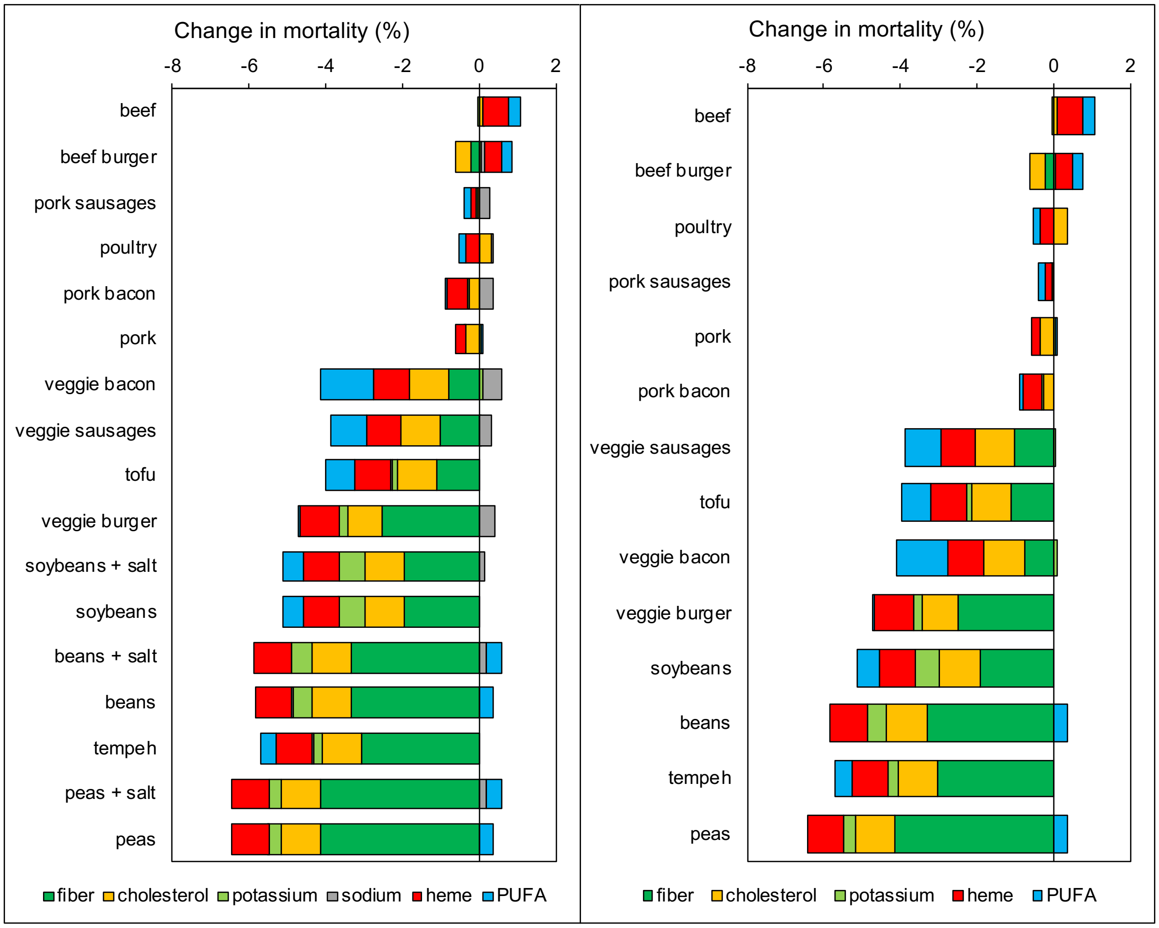

Supplementary environmental data and results

Table S13. Environmental footprints of meat and milk alternatives. The table provides an overview of global median values. The analysis uses regionalised values where possible, in particular when adapted from meta-analyses.

Category	Food product	Footprint per kg of product GHG ($kgCO_2$-eq)	land ($1000\ m^2$)	water ($1000\ m^3$)	Source
meat	beef	32.80	63.99	1.42	Poore and Nemecek (2018)[m]
	pork	11.62	12.62	0.97	Poore and Nemecek (2018)[m]
	poultry	5.76	6.59	0.39	Poore and Nemecek (2018)[m]
	beef burger	29.52	57.59	1.28	Poore and Nemecek (2018)[m]
	pork sausages	8.64	11.07	0.63	Carbon Trust (2022)[i], Ernst & Young (2020)[i]
	pork bacon	9.45	12.54	0.59	Carbon Trust (2022)[i]
meat alternative	veggie burger	2.06	1.80	0.09	Smetana et al (2015)[p], Fresan et al (2019)[p], Meija et al (2020)[p]
	veggie sausages	2.42	3.11	0.07	Meija et al (2020)[p], Carbon Trust (2022)[i], Ernst & Young (2020)[i]
	veggie bacon	3.02	3.30	0.03	Carbon Trust (2020)[i]
	tofu	2.48	3.12	0.14	Poore and Nemecek (2018)[m]
	tempeh	2.22	2.56	0.13	Ahnan-Winarno et al (2020)[p], Wiloso et al (2019)[p]; Poore and Nemecek (2018)[m]
	soybeans	1.27	5.30	0.22	Poore and Nemecek (2018)[m]
	beans	1.32	10.25	0.55	Poore and Nemecek (2018)[m]
	peas	0.76	6.17	0.36	Poore and Nemecek (2018)[m]
milk	milk	2.60	3.04	0.53	Poore and Nemecek (2018)[m]
	low-fat milk	2.60	3.04	0.53	Poore and Nemecek (2018)[m]
milk alternative	soy milk	0.98	0.66	0.03	Poore and Nemecek (2018)[m]
	oat milk	0.90	0.76	0.05	Poore and Nemecek (2018)[m]
	almond milk	0.70	0.50	0.37	Poore and Nemecek (2018)[m]
	rice milk	1.18	0.34	0.27	Poore and Nemecek (2018)[m]
	soybeans	1.27	5.30	0.22	Poore and Nemecek (2018)[m]
	oats	1.62	5.30	0.31	Poore and Nemecek (2018)[m]
	almonds	1.06	6.57	0.90	Poore and Nemecek (2018)[m]
	rice (brown)	3.28	1.97	2.06	Poore and Nemecek (2018)[m]

Notes: m: meta-analysis, p: peer-reviewed LCA, i: industry report. Values for beef burgers assume 90% beef content inferred from food composition data; values for tempeh inferred from difference to soybeans from peer-reviewed LCAs.

Table S14. Food-related environmental impacts in high-income countries for baseline diets and for the per-calorie replacement scenarios.

Category	Food product	GHG emissions ($MtCO_2$-eq)	Land use (1000 km^2)	Water use (km^3)
baseline		2,859,485	4,796,157	370,089
meat	beef	4,156,839	7,576,013	385,499
	pork	1,989,531	2,665,119	318,888
	poultry	2,113,043	2,864,359	334,692
	beef burger	3,206,129	5,529,214	343,775
	pork sausages	2,123,137	2,729,854	311,643
	pork bacon	1,980,670	2,565,659	297,430
meat alternative	veggie burger	1,821,769	2,248,358	282,591
	veggie sausages	1,777,962	2,288,949	278,065
	veggie bacon	1,784,199	2,258,222	274,436
	tofu	2,189,688	2,801,372	307,294
	tempeh	1,819,870	2,311,897	286,770
	soybeans	1,651,404	2,312,623	283,904
	beans	1,681,343	2,749,919	299,846
	peas	1,631,962	2,394,710	296,882
milk	milk	2,859,485	4,796,157	370,089
	low-fat milk	3,044,177	4,949,016	401,385
milk alternative	soy milk	2,669,628	4,584,051	287,611
	oat milk	2,572,298	4,562,826	291,391
	almond milk	2,958,282	4,804,786	619,890
	rice milk	2,660,183	4,443,930	356,867
	soybeans	2,357,349	4,528,758	285,168
	oats	2,373,226	4,525,439	289,228
	almonds	2,314,816	4,465,546	328,382
	rice	2,424,637	4,418,669	357,161

Fig. S2. Sensitivity analysis of changes in environmental impacts for replacing meat and milk with alternatives per calorie – with process-related emissions added to soybeans. For the analysis, an amount of 0.16 kgCO2-eq related to generic process-related activities (e.g., cooking) was added to the emissions footprints of soybeans.

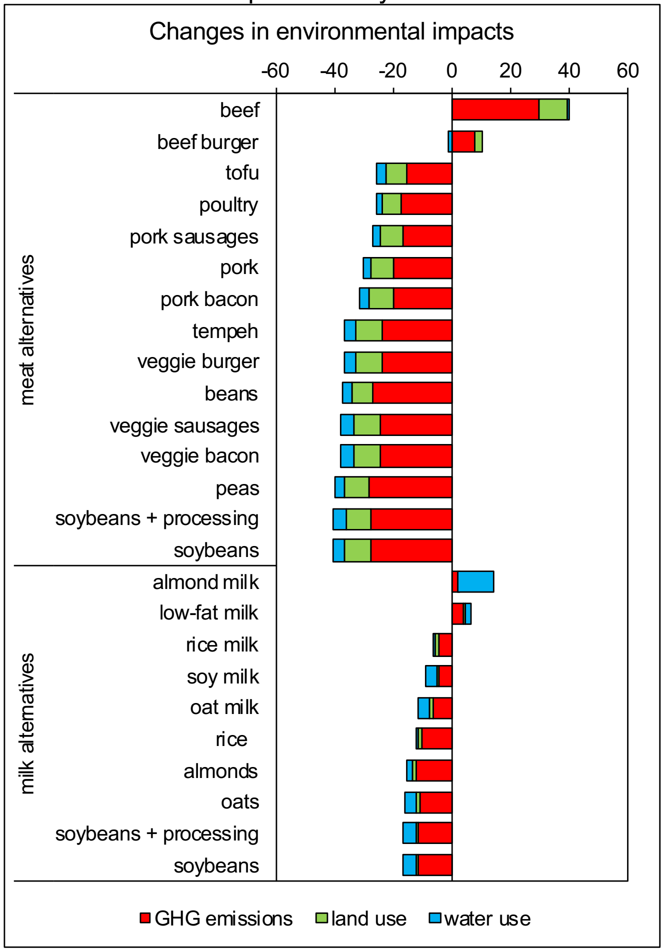

Supplementary cost data and results

Table S15. Prices of food products (USD per kg). The table provides an overview of the global values. The analysis uses regionalised values where possible, in particular when adapted from the International Comparison Program.

Category	Food product	Price (USD-2020) per kg			Source
		mean	low	high	
meat	beef	14.79	10.69	19.01	International Comparison Program
	pork	15.89	11.17	20.60	International Comparison Program
	poultry	4.09	3.10	5.23	International Comparison Program
	beef burger	10.82	8.21	13.36	Supermarket data (n=8)
	pork sausages	8.20	5.67	10.63	Supermarket data (n=24)
	pork bacon	13.34	7.78	18.64	Supermarket data (n=29)
meat alternative	veggie burger	14.66	9.59	19.54	Supermarket data (n=15)
	veggie sausages	11.55	7.86	15.11	Supermarket data (n=15)
	veggie bacon	26.57	21.09	31.93	Supermarket data (n=8)
	tofu	8.30	7.27	9.32	Supermarket data (n=8)
	tempeh	7.71	7.71	7.71	Supermarket data (n=1)
	soybeans	11.74	4.94	18.73	International Comparison Program
	beans	11.74	4.94	18.73	International Comparison Program
	peas	11.74	4.94	18.73	International Comparison Program
milk	milk	2.09	1.66	2.52	International Comparison Program
	low-fat milk	2.09	1.66	2.52	International Comparison Program
milk alternative	soy milk	2.45	1.70	3.18	Supermarket data (n=12)
	oat milk	2.77	2.48	3.06	Supermarket data (n=33)
	almond milk	2.77	2.40	3.14	Supermarket data (n=12)
	rice milk	3.02	2.94	3.11	Supermarket data (n=2)
	soybeans	11.74	4.94	18.73	International Comparison Program
	oats	2.73	1.75	3.66	International Comparison Program
	almonds	7.18	6.93	7.42	International Comparison Program
	rice (brown)	1.86	1.34	2.40	International Comparison Program

Table S16. Cost of diets (US$ per person per day) in high-income countries for baseline diets and in the per-calorie replacement scenarios.

Category	Food product	Cost of diets (US$ per day)		
		mean	low	high
baseline		8.92	6.07	11.98
meat	beef	10.69	6.25	15.37
	pork	8.45	5.86	11.13
	poultry	6.85	4.81	9.17
	beef burger	7.59	5.63	9.60
	pork sausages	6.91	5.00	8.86
	pork bacon	7.71	5.29	10.14
meat alternative	veggie burger	8.96	6.29	11.66
	veggie sausages	8.33	5.95	10.73
	veggie bacon	12.25	9.42	15.13
	tofu	8.91	7.06	10.82
	tempeh	7.10	5.71	8.56
	soybeans	5.70	4.26	7.21
	beans	5.79	4.34	7.31
	peas	5.79	4.34	7.31
milk	milk	8.92	6.07	11.98
	low-fat milk	8.92	6.07	11.98
milk alternative	soy milk	9.82	6.62	13.21
	oat milk	9.79	6.94	12.85
	almond milk	11.20	8.11	14.49
	rice milk	8.82	6.11	11.72
	soybeans	8.43	5.71	11.34
	oats	8.39	5.61	11.36
	almonds	8.57	5.88	11.46
	rice	8.39	5.60	11.39

Supplementary synthesis results

Fig. S3. Contribution of mitigation measures to simultaneously reduce environmental impacts below environmental limits (planetary boundaries); adapted from Springmann and colleagues (Nature 2018, Figure 4). The mitigation measures include dietary changes to flexitarian diets (FLX), technological improvements of medium (tech) and high ambition (tech+), halving of food loss and waste (w/2), and socioeconomic development with higher income growth and lower population growth (SSP1). Adapted from Springmann and colleagues (2018). We derived the weighing fractions by considering the percentage contribution of dietary changes to reducing GHG emissions (70%), land use (18%), water use (19%), and then normalised those contributions to sum to a total of 100%.

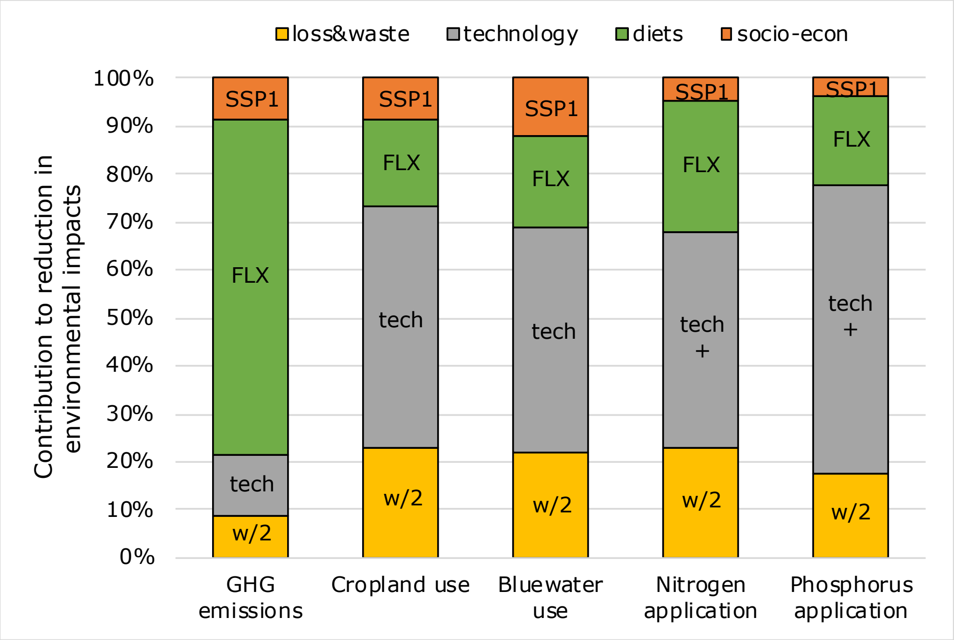

Table S17. Domain-specific performance scores derived in the multicriteria analysis of replacing meat and dairy per calorie in high-income countries.

Category	Food product	Performance scores across domains						weighted
		nutrition	mortality	GHGs	land use	water use	diet costs	average
meat	beef	0.31	0.00	0.00	0.00	0.00	0.24	0.13
	pork	0.00	0.22	0.86	0.92	0.60	0.58	0.50
	poultry	0.11	0.17	0.81	0.88	0.46	0.83	0.57
	beef burger	0.49	0.11	0.38	0.38	0.38	0.71	0.46
	pork sausages	0.14	0.16	0.81	0.91	0.66	0.82	0.59
	pork bacon	0.09	0.21	0.86	0.94	0.79	0.69	0.57
meat alternative	veggie burger	0.72	0.75	0.92	1.00	0.93	0.50	0.72
	veggie sausages	0.43	0.64	0.94	0.99	0.97	0.60	0.70
	veggie bacon	0.19	0.64	0.94	1.00	1.00	0.00	0.46
	tofu	0.44	0.71	0.78	0.90	0.70	0.51	0.62
	tempeh	0.57	0.95	0.93	0.99	0.89	0.79	0.82
	soybeans	1.00	0.86	0.99	0.99	0.91	1.00	0.97
	beans	0.82	0.91	0.98	0.91	0.77	0.99	0.93
	peas	0.90	1.00	1.00	0.97	0.80	0.99	0.96
milk	low-fat milk	0.65	0.00	0.00	0.00	0.65	0.81	0.42
milk alternative	soy milk	0.85	0.78	0.51	0.69	0.99	0.49	0.65
	oat milk	0.42	0.77	0.65	0.73	0.98	0.50	0.61
	almond milk	1.00	0.90	0.12	0.27	0.00	0.00	0.36
	rice milk	0.36	0.18	0.53	0.95	0.79	0.85	0.59
	soybeans	0.86	1.00	0.94	0.79	1.00	0.99	0.95
	oats	0.43	0.79	0.92	0.80	0.99	1.00	0.84
	almonds	0.50	0.80	1.00	0.91	0.87	0.93	0.85
	rice	0.00	0.23	0.85	1.00	0.78	1.00	0.66

Fig. S4. Changes in nutritional imbalances, mortality, food-related GHG emissions, land use, water use, and diet costs for a joint replacement analysis in which meat and dairy are simultaneously replaced per calorie by two combinations of meat and milk alternatives.

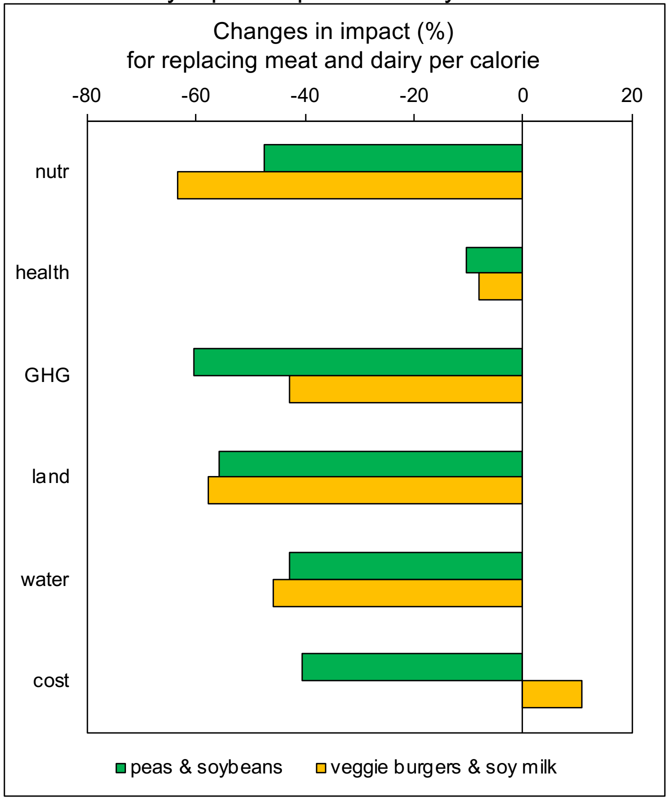

Fig. S5. Synthesis scores for different weighing schemes, including planetary-boundary weighing in the environmental analysis (*main analysis*), equal weights across environmental impacts (*environmental average*), and equal weights across all sub-domains (*equal subs*).

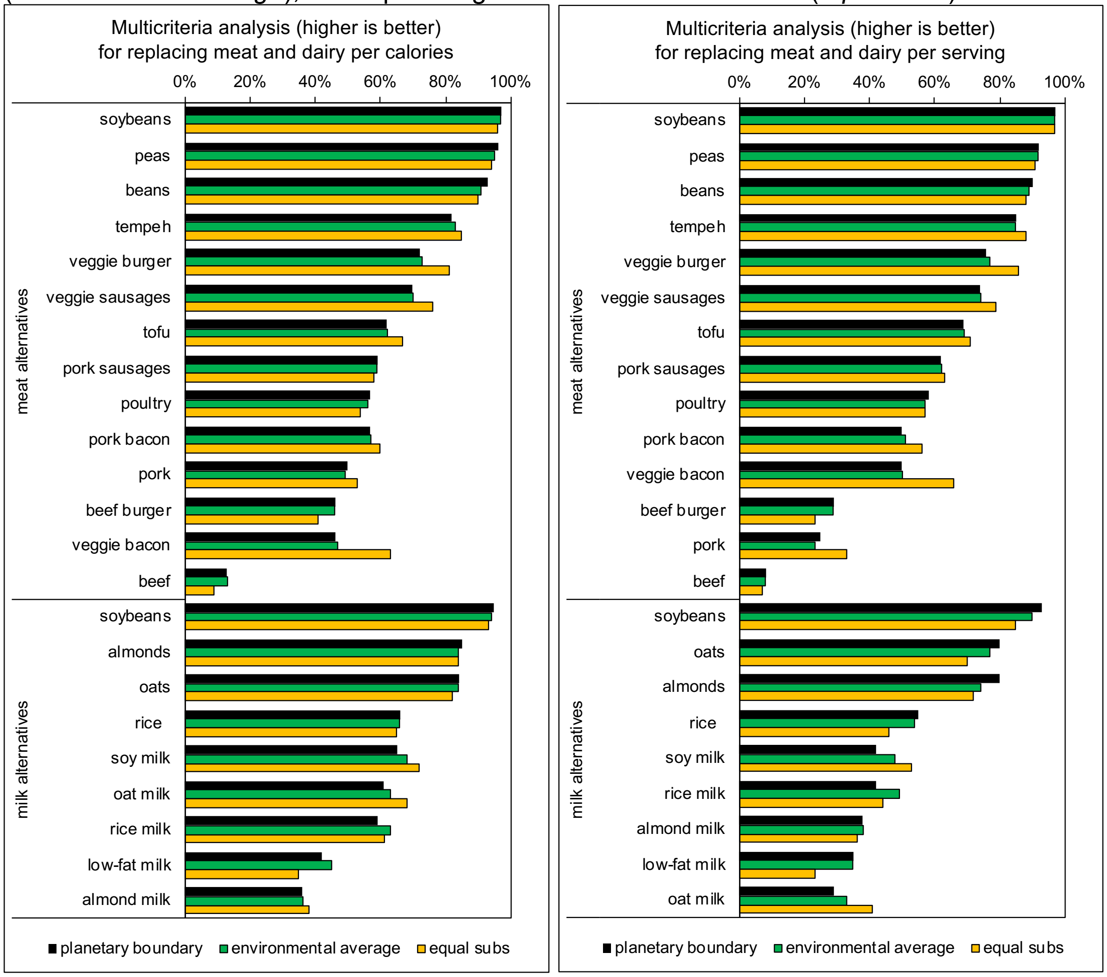

Fig S6. Multicriteria analysis of replacing meat and milk alternatives per calorie (left) and per serving (right) for different income regions, including high-income (HIC), upper middle-income (UMC), lower middle-income (LMC), and low-income (LIC) countries.

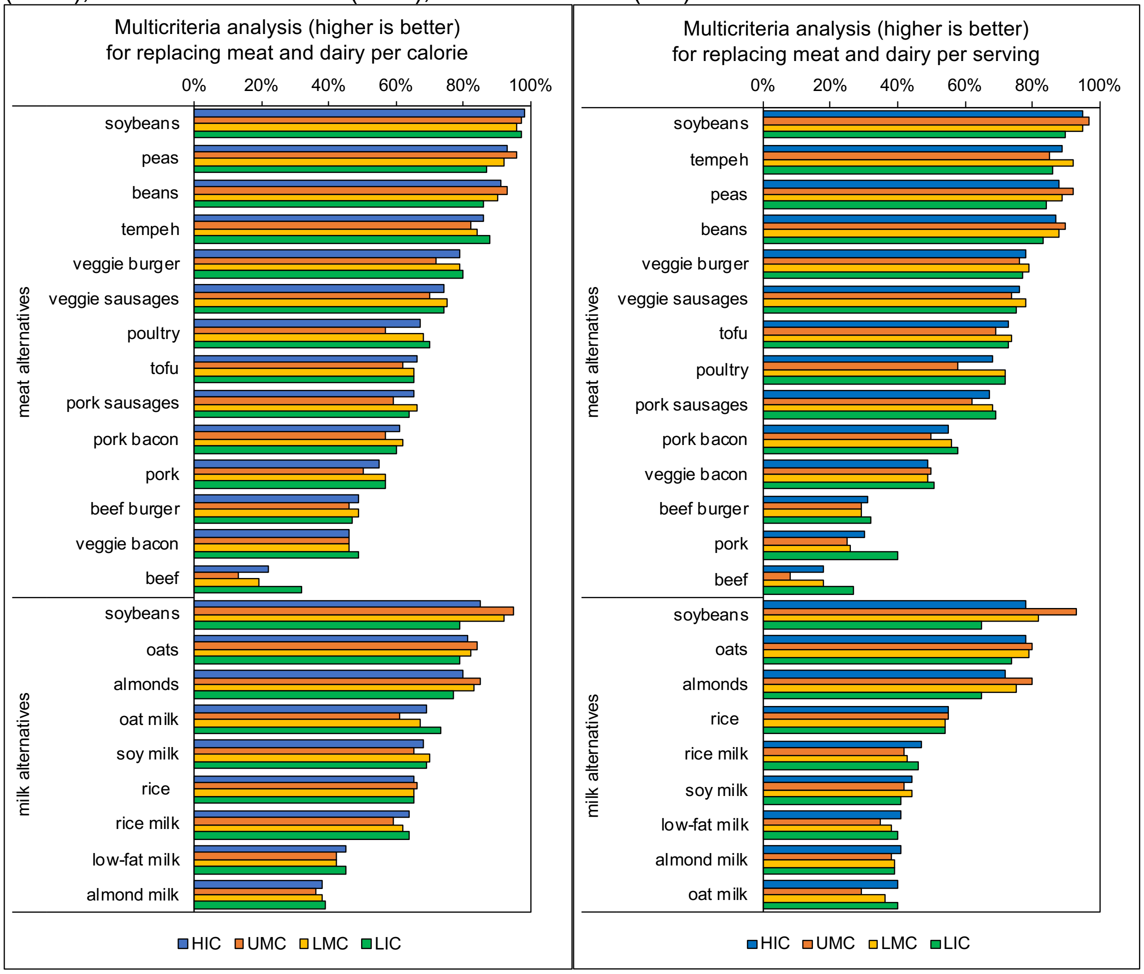

Fig. S7. Multicriteria analysis of replacing meat and milk per calorie by domain for different income regions, including high-income (HIC, A), upper middle-income (UMC, B), lower middle-income (LMC, C), and low-income (LIC, D) countries.

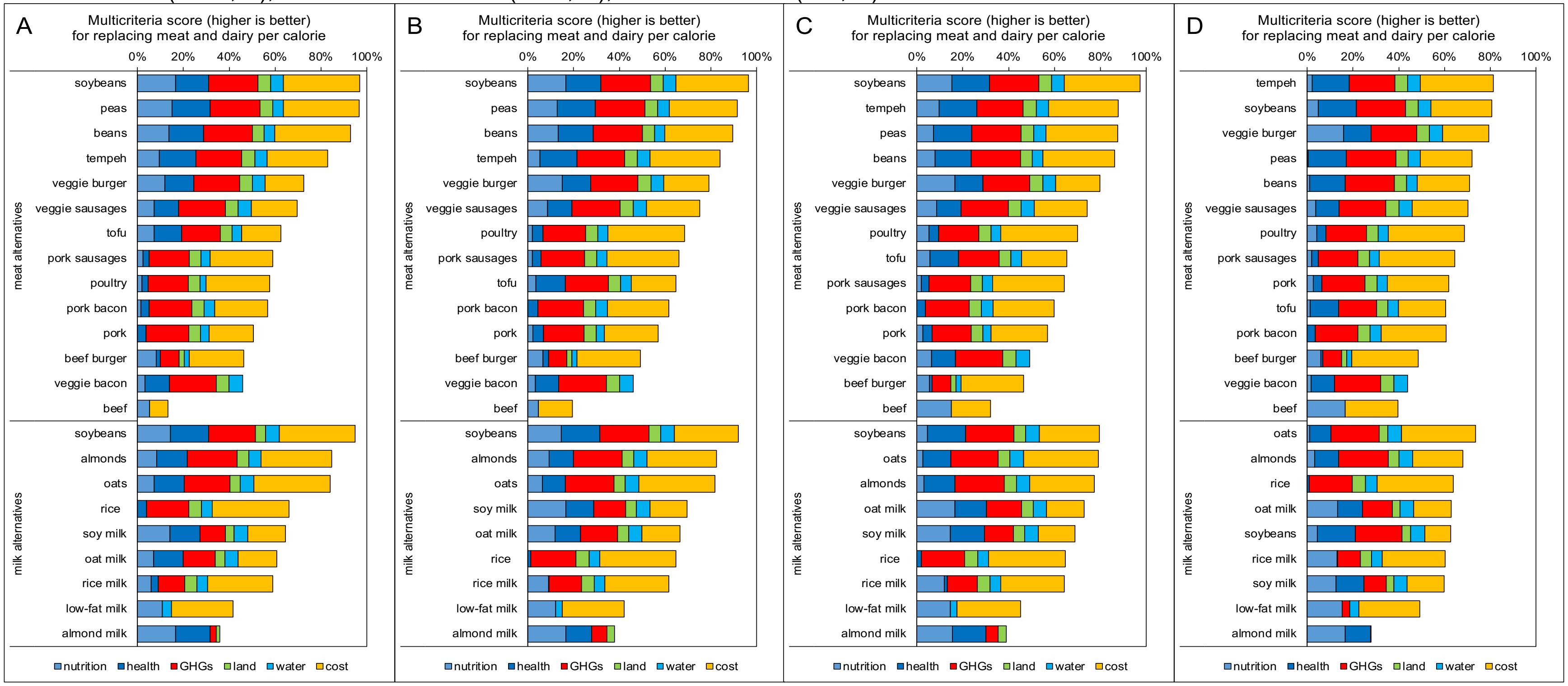

Table S18. Impact values for sensitivity analysis on cultivated beef. Values were selected to span the range of available estimates for environmental, cost, and nutritional impacts, specifying one base case (cultivated beef) that reflects current impacts and one best case (cultivated beef +) that reflects potential future improvements.

Food product	Parameter	Value	Source
Cultivated beef	GHG emissions	25.19 kgCO_2eq/kg	Review by Sinke et al (2023)
	Land use	8.47 m^2/kg	Review by Sinke et al (2023)
	Water use	0.86 m^3/kg	Review by Sinke et al (2023)
	Costs	442,244 USD/kg	Risner et al (2021)
	Nutrients	As beef bugers	Own assumption
Cultivated beef +	GHG emissions	1.89 kgCO_2eq/kg	Review by Sinke et al (2023)
	Land use	0.19 m^2/kg	Review by Sinke et al (2023)
	Water use	0.06 m^3/kg	Review by Sinke et al (2023)
	Costs	50.33 USD/kg	Garrison et al (2022), Humbird et al (2021
	Nutrients	As beef burgers but with half of saturated fats replaced by PUFAs	Own assumption

Fig. S8. Multi-criteria analysis (left) and percentage changes in impacts (right) with cultivated beef added to the analysis. The percentage increases in cost for cultivated beef (right) reach 796,117%. Because cultivated beef is not commercially viable at current technologies, its cost score was set to zero (left) without being used in the normalization procedure.

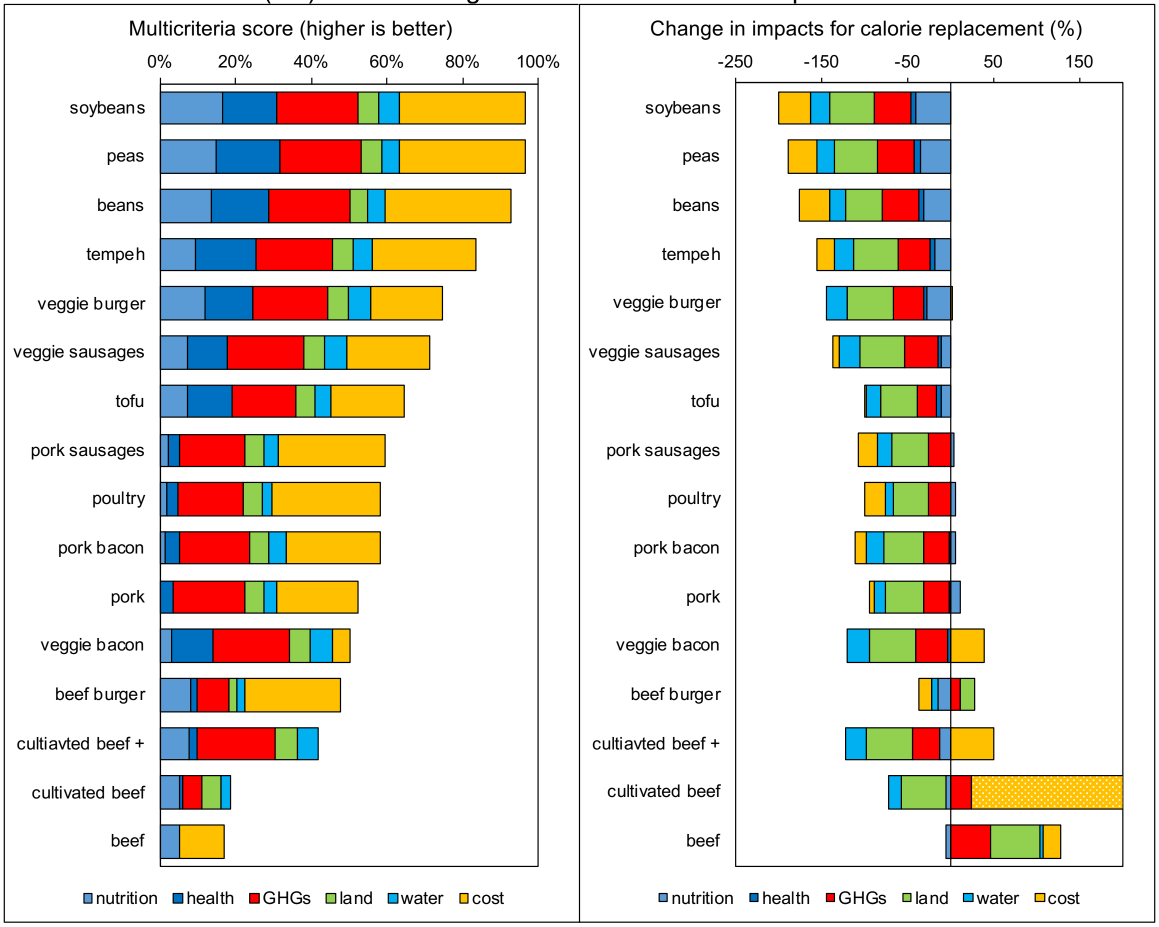

Supplement: Supplementary file 1 — Appendix 01 (PDF) [file pnas.2319010121.sapp.pdf]
